# Clinical Outcomes of Concomitant Use of Proton Pump Inhibitors and Dual Antiplatelet Therapy: A Systematic Review and Meta-Analysis

**DOI:** 10.3389/fphar.2021.694698

**Published:** 2021-08-02

**Authors:** Hongzhou Guo, Zhishuai Ye, Rongchong Huang

**Affiliations:** Cardiac Center/Division of Cardiovascular Diseases, Beijing Friendship Hospital, Capital Medical University, Beijing, China

**Keywords:** proton pump inhibitors, coronary artery disease, dual antiplatelet therapy, medication interaction, adverse cardiovascular events, meta-analysis

## Abstract

**Background:** The safety and efficacy associated with the use of proton pump inhibitors (PPIs) by patients with coronary artery disease receiving dual antiplatelet therapy (DAPT) remain unclear.

**Methods:** The evaluated outcomes included combined major adverse cardiovascular events (MACEs), myocardial infarction (MI), all-cause mortality, and gastrointestinal (GI) bleeding. A random effects meta-analysis, stratified by study design, was performed and heterogeneity was assessed using the I^2^ statistic.

**Results:** In total, 6 randomized controlled trials (RCTs) (6930 patients) and 16 observational studies (183,546 patients) were included. Analysis of RCTs showed that there were no significant differences in the incidences of MACEs (risk ratio [RR] = 0.89 [95% confidence interval (CI) = 0.75–1.05]), MI (RR = 0.93 [95% CI = 0.76–1.15]), and all-cause mortality (RR = 0.79 [95% CI = 0.50–1.23]) in the PPI groups vs. the non-PPI groups. Pooled data from observational studies revealed an inconsistent association between the use of each PPI subtype and the increased risks of MACEs during clopidogrel treatment. There was no increased risk of MACEs or all-cause mortality associated with the use of PPIs (as a class) and other P2Y_12_ inhibitors. Both the RCTs and observational studies revealed that the use of PPIs significantly reduced the risks of GI bleeding.

**Conclusion:** The use of PPIs was associated with a reduced risk of GI bleeding in patients treated with DAPT after percutaneous coronary intervention or acute coronary syndrome. There was no clear evidence of an association between the use of PPIs and adverse cardiovascular events.

Clinical Trial Registration: identifier [CRD42020190315]

## Introduction

Dual antiplatelet therapy (DAPT) with an oral P2Y_12_ receptor inhibitors and aspirin constitute the foundation antiplatelet strategy after percutaneous coronary intervention (PCI) or acute coronary syndrome (ACS) (Neumann et al., 2019). The main drawback of DAPT remains an increased incidence of bleeding events that can lead to discontinuation of therapy and most importantly, increased mortality (Ducrocq et al., 2015). The gastrointestinal (GI) tract is a common source of bleeding in response to DAPT (Kazi et al., 2015). The use of proton pump inhibitors (PPIs) can reduce the risk of peptic ulceration by lowering the acidity in the gastric and duodenal lumens, and may also reduce the severity of GI bleeding by enhancing the stability of clots (Scheiman, 2013). Therefore, PPIs combined with DAPT presents a feasible and biologically plausible strategy to reduce GI bleeding, thereby reducing the risk of ischemic events. However, the potential negative effect of PPIs on cardiovascular (CV) outcomes remains controversial.

Several randomized controlled trials (RCTs) and observational studies have had mixed results as to whether the proposed metabolic interactions of PPIs and clopidogrel are associated with an increased risk of poor CV outcomes (Ho et al., 2009; Bhatt et al., 2010; Kreutz et al., 2010; Kwok and Loke, 2010; Kwok et al., 2013; Jensen et al., 2017). Furthermore, these risks were also found in the general population, indicating that the risk of poor CV outcomes associated with the use of PPIs combined with clopidogrel may be partly or directly conferred by PPIs (Shah et al., 2015; Wang et al., 2017).

Previous studies have mainly focused on the metabolic interactions between PPIs and clopidogrel, but failed to address the influence of PPIs with DAPT in coronary artery disease. Furthermore, the efficiency of PPIs to reduce GI bleeding in patients after DAPT has yet to be systematically evaluated. Within this framework, the aim of this comprehensive systematic review and meta-analysis was to assess the efficacy and safety of PPIs with DAPT for the treatment of coronary artery disease.

## Methods

### Data Sources and Search Strategy

The systematic review and meta-analysis were conducted in accordance with the Preferred Reporting Items for Systematic Reviews and Meta-Analyses (PRISMA) statement (Supplementary Table S1) (Hutton et al., 2015). The study protocol is registered with the International Prospective Register of Systematic Reviews (CRD42020190315). Relevant studies were retrieved from the PubMed, Cochrane Library, EMBASE, and Web of Science electronic databases, as well as the ClinicalTrials.gov website, from inception to May 2020. The complete search strategy is outlined in Supplementary Table S2. The reference lists of relevant articles were reviewed for identification of potential eligible studies that might have been missed.

### Study Selection

The study selection process, which was independently performed by two reviewers (H.G. and Z.Y.), included screening of all titles, abstracts, and full texts in order to identify potentially eligible studies. Any discrepancy in the assessments was resolved by discussion with a third reviewer (R.H).

The included articles were limited to RCTs and observational studies published in English that compared the efficacy or safety of PPIs versus a placebo or no PPI in patients after PCI or ACS. The observational studies must have reported the effects of individual PPIs separately on CV outcomes and all-cause mortality for those treated with clopidogrel. Studies comparing PPI to other anti-GI bleeding regimens (i.e., histamine-2 receptor antagonists) were excluded. Observational studies that did not provide adjusted effect estimates on the outcomes of interest were also excluded. The dose and duration of PPI therapy were not restricted, although sensitivity analysis based on the duration of exposure was conducted. For GI bleeding, the types of PPIs and P2Y_12_ inhibitors were not restricted.

The outcomes included major adverse cardiovascular events (MACEs), myocardial infarction (MI), all-cause mortality, GI bleeding, and upper gastrointestinal (UGI) bleeding. When extracting data on GI bleeding, data pertaining to UGI bleeding were also included. The definitions of outcomes are presented in Supplementary Table S3.

### Data Abstraction and Quality Assessment

Two reviewers (H.G. and Z.Y.) independently abstracted the data from each eligible study with adjudication by a third reviewer (R.H.). As opposed to contacting the original authors, studies with insufficient or unavailable pooled data were excluded. The abstracted data included baseline characteristics of the studies and participants, descriptions of the interventions and control conditions, information for the assessment of the risk of bias, outcomes, and methods used to address confounding factors. Two reviewers (H.G. and Z.Y.) independently appraised the risk of bias and any disagreement was resolved by consensus with a third reviewer. The quality of the observational studies in regard to participant selection, population comparability, and outcome/exposure assessment was appraised using the Newcastle–Ottawa Scale (NOS) (Wells et al., 2014). The Cochrane Collaboration tool was used to evaluate the quality of the RCTs (Higgins and Altman, 2008).

### Data Synthesis and Analysis

Review Manager 5.4 software [Nordic Cochrane Center, Rigshospitalet, Denmark; (http://ims.cochrane.org/revman)] was used to calculate the risk ratio (RR) and 95% confidence interval (CI) of the clinical endpoints with an inverse variance random effects method. Data from the RCTs and observational studies were pooled independently. Reported odds ratios (ORs) were converted to RR according to the Cochrane Handbook for Systematic Reviews of Interventions: RR = OR/[1–ACRx (1–OR)]. As in previous studies, similarity between the hazard ratios (HRs) and RRs was assumed because the clinical outcomes in the present study were uncommon events (Siller-Matula et al., 2010; Kwok et al., 2011; Lambert et al., 2015) and sensitivity analyses were performed to estimate the CV outcomes. Statistical heterogeneity was assessed using the Cochran Q test and Higgins I^2^ test, with a probability (*p*) value of <0.10 and I^2^ statistic of >50% indicating significant heterogeneity (Higgins et al., 2003). Publication bias was estimated if the numbers of outcomes in the studies were sufficient.

## Results

### Search Results and Characteristics

**FIGURE 1:**
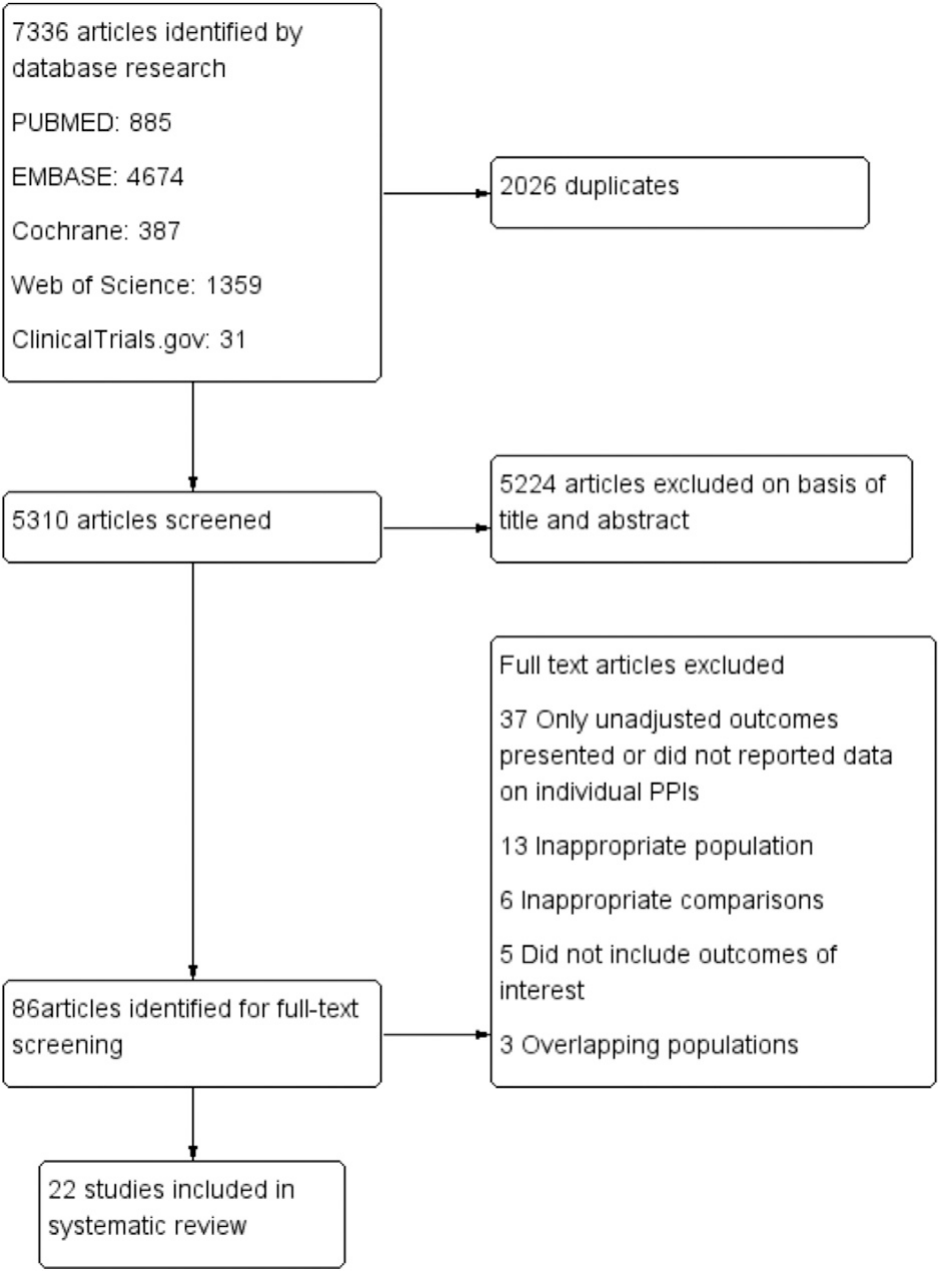
Flow diagram of study selection process.

The study screening process and reasons for exclusion are presented in the form of a PRISMA flow chart presented in Figure 1. Of 7336 articles, 5,310 were screened after removal of duplicate publications. After screening of the titles and abstracts, the full texts of 86 articles were assessed, of which 22 met the inclusion criteria (Ng et al., 2008; Gao et al., 2009; Ho et al., 2009; Juurlink et al., 2009; O'Donoghue et al., 2009; Rassen et al., 2009; Bhatt et al., 2010; Charlot et al., 2010; Kreutz et al., 2010; Ray et al., 2010; Ren et al., 2011; Simon et al., 2011; Wu et al., 2011; Goodman et al., 2012; Schmidt et al., 2012; Jiang et al., 2013; Hokimoto et al., 2014; Yan et al., 2016; Jensen et al., 2017; Hoedemaker et al., 2019; Sehested et al., 2019 and Zhang et al., 2019). The lack of data on individual PPIs and only unadjusted outcomes presented were the most common reasons for exclusion. These 22 articles, which included six RCTs (Gao et al., 2009; Bhatt et al., 2010; Ren et al., 2011; Wu et al., 2011; Jensen et al., 2017; Zhang et al., 2019) and 16 observational studies (Ng et al., 2008; Ho et al., 2009; Juurlink et al., 2009; O'Donoghue et al., 2009; Rassen et al., 2009; Charlot et al., 2010; Kreutz et al., 2010; Ray et al., 2010; Simon et al., 2011; Goodman et al., 2012; Schmidt et al., 2012; Jiang et al., 2013; Hokimoto et al., 2014; Yan et al., 2016; Hoedemaker et al., 2019; Sehested et al., 2019), enrolled a total of 190,476 patients. Data retrieved from the RCTs and observational studies were pooled separately. Due to the limited number of RCTs, PPIs were assessed as a class when investigating the effects of these drugs co-administered with DAPT. The 16 observational studies comprised 12 cohort studies (Ng et al., 2008; Ho et al., 2009; Rassen et al., 2009; Charlot et al., 2010; Kreutz et al., 2010; Ray et al., 2010; Simon et al., 2011; Schmidt et al., 2012; Hokimoto et al., 2014; Yan et al., 2016; Hoedemaker et al., 2019; Sehested et al., 2019), two case-control studies (Juurlink et al., 2009; Jiang et al., 2013), and two post-hoc analyses of RCTs (O'Donoghue et al., 2009; Goodman et al., 2012). Of the 16 observational studies, nine reported the risk of CV outcomes in response to clopidogrel plus individual PPIs (Ho et al., 2009; Juurlink et al., 2009; O'Donoghue et al., 2009; Rassen et al., 2009; Kreutz et al., 2010; Ray et al., 2010; Simon et al., 2011; Schmidt et al., 2012; Hokimoto et al., 2014), and three analyzed PPIs as a class plus other P2Y_12_ inhibitors (O'Donoghue et al., 2009; Goodman et al., 2012; Yan et al., 2016). The main characteristics of the individual studies included in this systematic review are summarized in Table 1.

**TABLE 1:** Study characteristics.

Study (year)	Study design; Country (time period)	Population; No. patients	Age (years); Males (%)	PPIs	P2Y_12_ inhibitors	Follow-up	Ascertainment of exposure	Ascertainment of outcome	Ajustment method	Adjustment for potential confounders
Bhatt et al. (2010)	Double blind randomised controlled trial; Multinational (January 2008 to December 2008)	ACS or PCI	69; 68	Omeprazole	Clopidogrel	Median time 106 days	Randomised intervention	Evaluate by independent committee	Not necessary	Not necessary
		Total (n = 3,761)								
		PPI (n = 1876)								
		No PPI (1885)								
Gao (2009)	Double blind randomised controlled trial; China (January 2003 to December 2007)	AMI	58; 53	Omeprazole	Clopidogrel	14 days	Randomised intervention	Not reported	Not necessary	Not necessary
		Total (n = 237)								
		PPI (n = 114)								
		No PPI (n = 123)								
Jensen (2017)	Randomised controlle d trial; Denmark (April 2011 to May 2013)	First-time PCI	64.7; 74	Pantoprazole	Clopidogrel	1 year	Randomised intervention	Not reported	Not necessary	Not necessary
		Total (n = 2009)								
		PPI (n = 997)								
		No PPI (n = 1,012)								
Ren (2011)	Randomised controlled trial; China (2011)	ACS with PCI	62; 72	Omeprazole	Clopidogrel	1 month	Randomised intervention	Not reported	Not necessary	Not necessary
		Total (n = 172)								
		PPI (n = 86)								
		No PPI (n = 86)								
Wu (2011)	Double blind randomised controlled trial; China (May 2008 to April 2010)	ACS with high risk of GI bleeding	Most≥75; 74	Pantoprazole	Clopidogrel	Median time 12 days	Randomised intervention	Evaluate by investigators	Not necessary	Not necessary
		Total (n = 665)								
		PPI (n = 333)								
		No PPI (n = 332)								
Zhang (2019)	Double blind randomised controlled trial; China (July 2015 to December 2016)	AMI with PCI	60；70	Omeprazole	Ticagrelor	6 months	Randomised intervention	Not reported	Not necessary	Not necessary
		Total (n = 86)								
		PPI (n = 43)								
		No PPI (n = 43)								
Charlot et al. (2010)	Observational cohort study; Denmark (2000–2006)	MI; Total (n = 24,702)	66; 59	Omeprazole; Esomeprazole	Clopidogrel	1 year	National Patient Registry	ICD-9 codes from the validated National Patient Registry	Cox proportional hazards models with propensity score	Age,sex, PCI, income, concomitant medical treatment, comorbid conditions
		PPI (n = 6,753)		Lansoprazole						
		No PPI (n = 17,949)		Pantoprazole						
Goodman et al. (2012)	Post hoc analysis of randomised controlled trial; Multinational (October 2006 to July 2008)	ACS	Median Age 62 to 63; 72	Omeprazole	Clopidogrel Ticagrelor	1 year	PPI use was at the discretion of the patient’s physician, and was identified at follow up	End points were adjudicated by an independent clinical events committee	Cox proportional hazards	Sex, race, region, peptic ulcer history, previous MI, systolic blood pressure, heart rate, hemoglobin, creatinine clearance, concomitant medical treatment, index event
		Total (n = 9,325)		Pantoprazole						
		PPI (n = 3,284)		Esomeprazole						
		No PPI (n = 6,041)		Lansoprazole						
				Rabeprazole						
Ho et al. (2009)	Observational cohort study and case-control study; US (October 2003 to January 2006)	ACS	67; 98.5	Omeprazole	Clopidogrel	Median 521 days	Pharmacy prescription records from Veterans Health Administration peer review program database	Chart review, vital status file, ICD-9 codes using Veterans Administration database	Multivariable adjustments	Age, diabetes, previous MI, PCI, CABG, heart failure, cerebrovascular disease, PVD, cancer, COPD, renal disease, dementia, liver disease, various medications
		Total (n = 8,205)		Lansoprazole						
		PPI (n = 5,244)		Pantoprazole						
		No PPI (n = 2,961)		Rabeprazole						
Hoedemaker (2018)	Observational cohort study; Dutch (2010–2014)	ACS; Total (n = 3,440)	65; 70	Not reported	Clopidogrel Ticagrelor	1 month	Drug prescriptions from medical records and was identified at follow up	Telephone interviews and patients records	Propensity-adjusted	Age, sex, smoking, diabetes, family history of CAD, hypercholesterolaemia, hypertension, stroke, MI, CABG, PCI, MI, eGFR, concomitant medication, year of admission
		PPI (n = 1974)								
		No PPI (n = 1,466)								
Hokimoto et al. (2014)	Observational cohort study; Japaese (December 2008 to January 2010)	PCI	69; 67	Rabeprazole	Clopidogrel	18 months	Rabeprazole use was at the discretion of the treating physician	Hospital records and contact with patients and their families	Multivariate Cox proportional hazards analysis	Age, diabetes, smoking, BMI, eGFR, multivessel disease, EF, CYP2C19 genotype
		Total (n = 174)								
		PPI (n = 50)								
		No PPI (n = 124)								
Jiang et al. (2013)	Case-control study; China (January 2008 to January 2011)	PCI; Total (n = 2,680)	71; 66	Omeprazole	Clopidogrel	1 year	Cardiology and department of gastroenterology databases	Cardiology and department of gastroenterology databases	Multivariate logistic regression	Age, gender, smoking, drinking status, hypertension, diabetes, previous peptic ulcer, previous GI bleeding
		PPI (n = 1,570)		Esomeprazole						
		No PPI (n = 1,110)		Lansoprazole						
Juurlink et al. (2009)	Nested case–control study; Canada (April 2002 to December 2007)	AMI	Median age 77; 54%	Pantoprazole any other PPI	Clopidogrel	1 month	Prescription records from the Ontario Public Drug Program	ICD codes from Canadian Institute for Health Information DisCharge Abstract database	Multivariate Logistic regression	Age, sex, income, Charlson comorbidity index, hospitalization, diabetes with complications, dysrhythmias, pulmonary edema, cardiogenic shock, acute renal insufficiency, chronic renal insufficiency, congestive heart failure, cerebrovascular disease
		Total (n = 2,791)								
		PPI (n = 1,026)								
		No PPI (n = 1765)								
Kreutz et al. (2010)	Observational cohort study; United States (October 2005 to September 2006)	PCI	66; 69	Omeprazole	Clopidogrel	1 year	Prescription records from the medical and pharmacy claims database	ICD-9-CM codes and Current Procedural Terminology, Fourth Edition codes from Medco Health Solutions Inc	Cox proportional hazards models	Age, sex, hospitalization, stent, diabetes, hypertension, heart failure, chronic kidney disease, dyslipidemia, concomitant medication
		Total (n = 16,690)		Esomeprazole						
		PPI (n = 6,828)		Lansoprazole						
		No PPI (n = 9,862)		Pantoprazole						
				Rabeprazole						
Ng et al. (2008)	Observational cohort study; China (January 2002 to December 2006)	ACS	72; 67	Omeprazole	Clopidogrel	7 days	Clinical Management System database	ICD codes and clinical records	Multiple logistic regression analysis	Age, previous peptic ulcer, mechanical ventilation, corticosteroid therapy, hospitalization
		Total (n = 626)		Esomeprazole						
		PPI (n = 336)		Pantoprazole						
		No PPI (n = 290)		Lansoprazole						
				Rabeprazole						
O’Donoghue (2009)	Post hoc analysis of randomised controlled trial; Multinational (November 2004 to July 2007)	ACS with PCI	61；74	Omeprazole	Clopidogrel Prasugrel	15 months	PPI use was at the discretion of the treating physician, and was identified at follow up	Not reported	Multivariable Cox proportional model with Propensity score	Age, sex, race, hypertension, hypercholesterolaemia, diabetes, smoking, index event, MI, CABG, stroke, transient ishaemic attack, CAD, PAD, heart failure, peptic ulcer disease, carotid or vertebral artery disease, creatinine clearance, stents, multivessel percutaneous intervention, concomitant medication, BMI, Hgb, systolic blood pressure, heart rate
		Total (n = 13,608)		Esomeprazole						
		PPI (n = 4,529)		Lansoprazole						
		No PPI (n = 9,079)		Pantoprazole						
Rassen et al. (2009)	Observational cohort study; US, Canada (January 2001 to December 2005)	ACS or PCI	76; 48	Omeprazole	Clopidogrel	6 months	Prescription records from 3 insurance databases	MI recorded in insurance database, death was assessed through vital statistics and government agencies	Cox proportional hazards regression	Age, gender, race, calendar year, index event, hospitalization, medications, medical service use
		Total (n = 18,565)		Esomeprazole						
		PPI (n = 3,996)		Lansoprazole						
		No PPI (n = 14,569)		Pantoprazole						
				Rabeprazole						
Ray et al. (2010)	Observational cohort study; US (January 1999 to December 31, 2005)	ACS or PCI	60.5; 50	Omeprazole; Esomeprazole	Clopidogrel	1 year	Prescription record from Tennessee Medicaid program	Admissions data and death certifificates	Cox regression model with propensity score	Age, sex, health insurance, race, calendar year, CABG, stent, propensity score
		Total (n = 20,596)		Lansoprazole						
		PPI (n = 7,593)		Pantoprazole						
		No PPI (n = 13,003)		Rabeprazole						
Schmidt et al. (2012)	Observational cohort study; Denmark (January 2002 to June 2005)	PCI; Total (n = 13,001)	Median age 64； 72	Omeprazole; Esomeprazole	Clopidogrel	1 year	National medical databases	Relevant records review by cardiac specialists	Cox proportional hazards regression	Age, gender, diabetes, hypertension, obesity, concomitant medication
		PPI (n = 2,742)		Lansoprazole						
		No PPI (n = 10,259)		Pantoprazole						
Sehested et al. (2019)	Observational cohort study; Danish (January 2003 to December 2014)	AMI; Total (n = 46,301)	Median age 68; 68	Not reported	Not reported	1 year	Danish nationwide registries	ICD-10 codes and Therapeutic Chemical codes	Cox regression models	Age, calendar year, sex, comorbidities, concomitant medication
		PPI (n = 8,980)								
		No PPI (n = 37,321)								
Simon et al. (2011)	Observational cohort study; France (October 2005 to November 2005)	MI	64; 72	Omeprazole	Clopidogrel	1 year	Computerized report forms for individual patients	Not reported	Multivariate Cox proportional model	Sex, GRACE score, hypertension, diabetes, smoking, hyperlipidemia
		Total (n = 2,353)		Esomeprazole						
		PPI (n = 1,453)		Lansoprazole						
		No PPI (n = 900)		Pantoprazole						
Yan et al. (2016)	Observational cohort study; Multinational (2003–2014)	ACS with PCI	Median age 61.25 to 66.22; 77	Not reported	Clopidogrel Ticagrelor	1 year	Database constructed by merging the individual databases from the remaining 15 centers	Obtained by telephone or face-to-face talk, and reviewed by the medical records of the index events	Cox model with propensity score	Age, sex, diabetes, hypertension, PAD, history of cancer, serum creatinine at admission and hemoglobin at admission
		Total (n = 489)								
		PPI (n = 351)								
		No PPI (n = 138)								

ACS, acute coronary syndrome; AMI, acute myocardial infarction; BMI, body mass index; CABG, coronary artery bypass graft; CAD, coronary artery disease; COPD, chronic obstructive pulmonary disease; CYP2C19, Cytochrome P450 2C19; EF, ejection fraction; eGFR, estimated glomerular filtration rate; GI, gastrointestinal; GRACE, Global Registry of Acute Coronary Events; Hgb, hemoglobin; ICD, International Classification of Diseases; MI, myocardial infarction; PAD, peripheral arterial disease; PCI, percutaneous coronary intervention; PPI, proton pump inhibitor; PVD, peripheral vascular disease.

### Risk of Bias and Publication Bias

Generally, RCTs have low or unclear risks of random sequence generation, concealment of allocation, and incomplete outcome data. The study by Jensen et al. (2017) was judged to have a high risk of blinding of participants and personnel because there was no blinding or placebo control, although the authors suggested that these shortcomings had no significant impact on the outcomes as the patients in the control group were not informed about their randomization status or risk assessment. The study by Ren et al. (2011) was deemed as high risk of selective reporting given that the reported outcomes were insufficient. Two post-hoc analyses of RCTs (O'Donoghue et al., 2009; Goodman et al., 2012) were assessed as observational studies for quality assessment. In all of the included observational studies, the analyses were adjusted to reduce the effects of potential confounding factors and had a low risk of bias. The risks of bias of 22 studies are summarized in Supplementary Table S4. Publication bias was not assessed given the insufficient numbers of outcomes in the included studies.

### Major Adverse Cardiovascular Events

**FIGURE 2:**
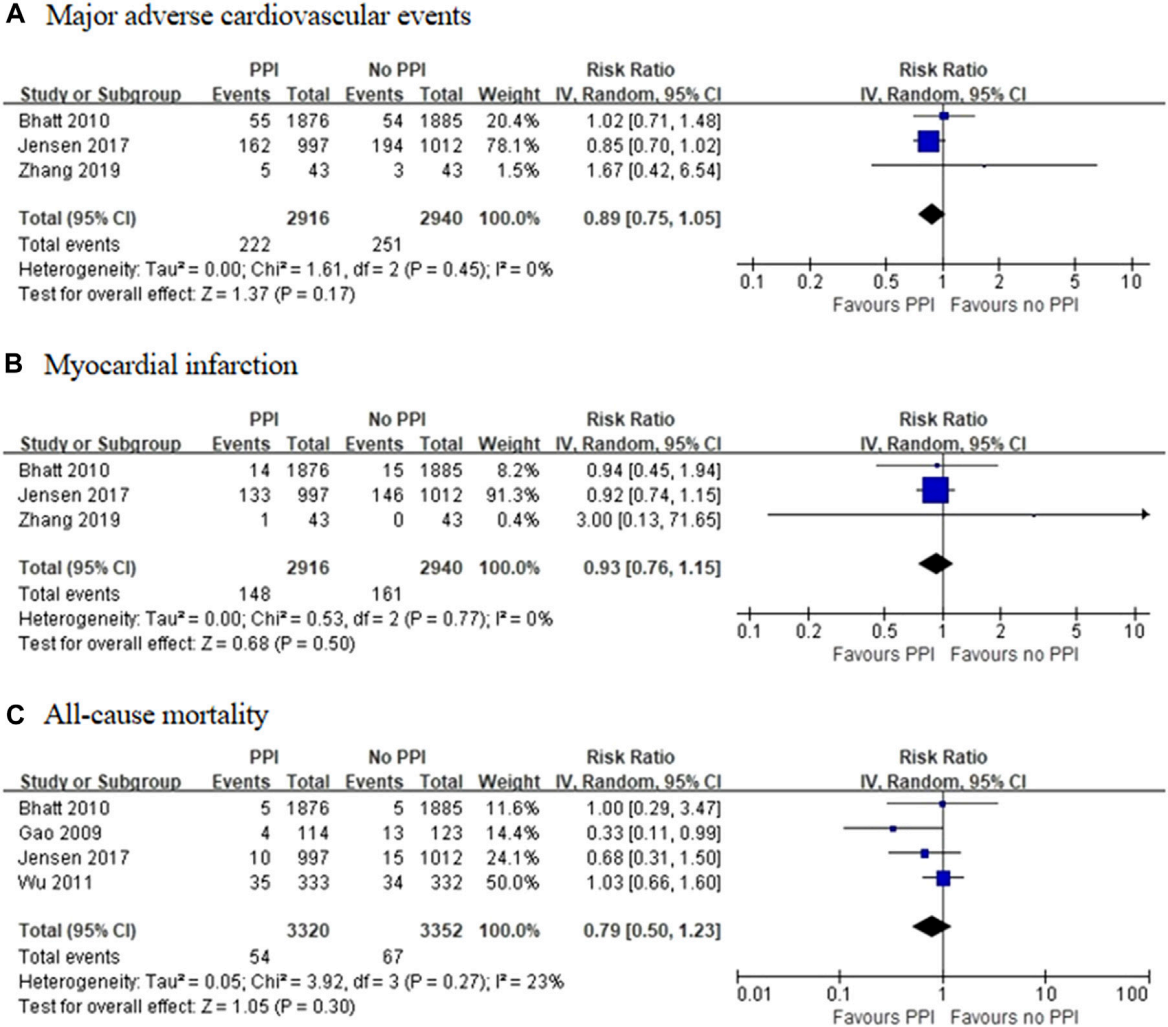
Meta-analysis of randomised controlled trials of major adverse cardiovascular events **(A)**, myocardial infarction **(B)** and all-cause mortality **(C)** with dual antiplatelet therapy and proton pump inhibitor use.

**FIGURE 3:**
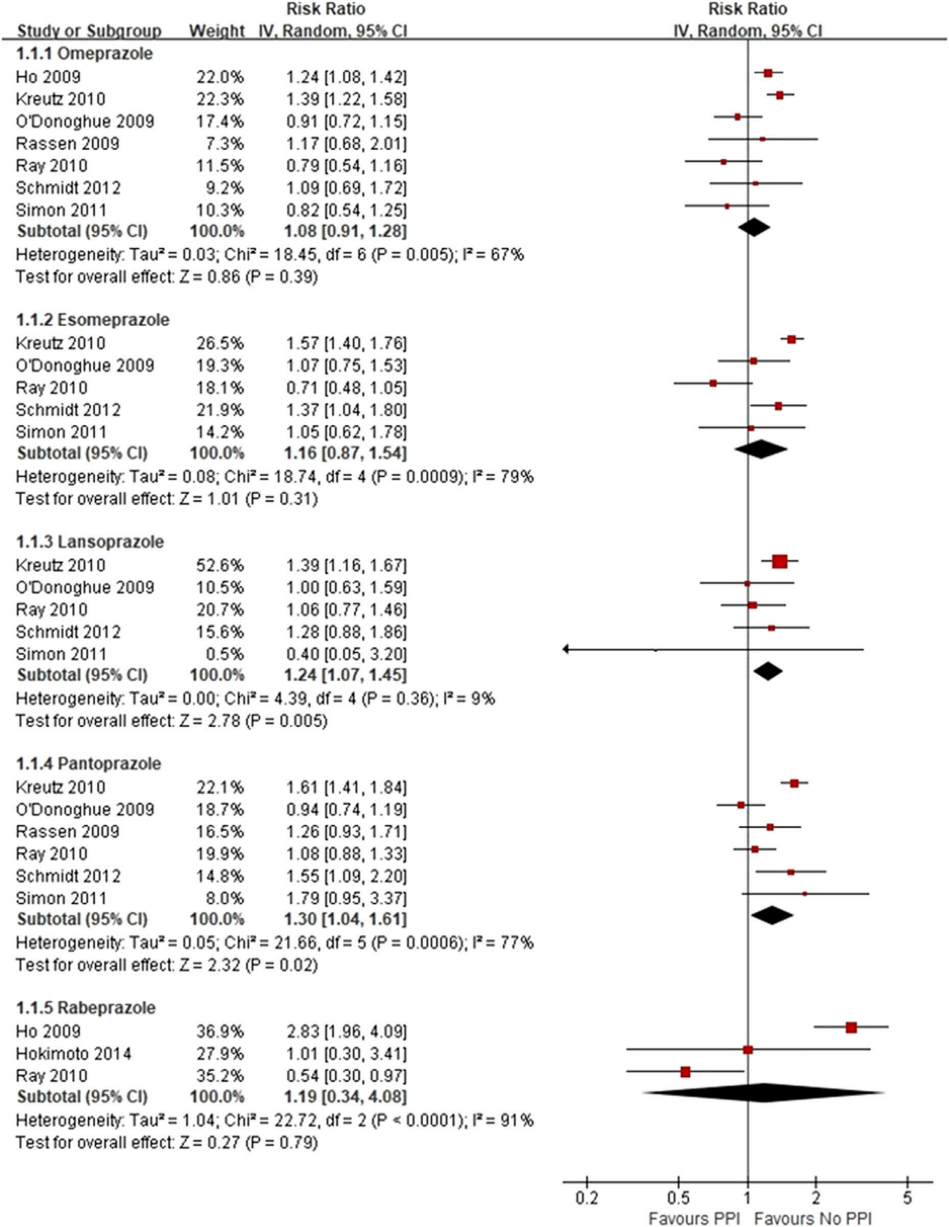
Meta-analysis of observational studies of major adverse cardiovascular events with clopidogrel and individual proton pump inhibitor use.

Three RCTs, which included 5,856 patients (Bhatt et al., 2010; Jensen et al., 2017; Zhang et al., 2019), reported no significant difference in the incidence of MACEs between the experimental and control groups (RR = 0.89 [95% CI = 0.75–1.05], *p* = 0.17, I^2^ = 0%; Figure 2). Of the 16 observational studies, eight reported the risk of MACEs with clopidogrel plus individual PPIs (Ho et al., 2009; O'Donoghue et al., 2009; Rassen et al., 2009; Kreutz et al., 2010; Ray et al., 2010; Simon et al., 2011; Schmidt et al., 2012; Hokimoto et al., 2014). As compared with regimens with no PPIs, the use of lansoprazole was associated with an increased risk for MACEs (RR = 1.24 [95% CI = 1.07–1.45], *p* = 0.005, I^2^ = 9%), as was pantoprazole (RR = 1.30 [95% CI = 1.04–1.61], *p* < 0.001, I^2^ = 77%), but not omeprazole (RR = 1.08 [95% CI = 0.91–1.28], *p* = 0.39, I^2^ = 67%), esomeprazole (RR = 1.16 [95% CI = 0.87–1.54], *p* = 0.31, I^2^ = 79%), and rabeprazole (RR = 1.19 [95% CI = 0.34–4.08], *p* = 0.79, I^2^ = 91%) when combined with clopidogrel (Figure 3). Meta-analysis of three observational studies revealed no increased risk of MACEs when PPIs were assessed as a class co-administered with other P2Y_12_ inhibitors (RR = 1.13 [95% CI = 0.91–1.42], *p* = 0.26, I^2^ = 59%) (O'Donoghue et al., 2009; Goodman et al., 2012; Yan et al., 2016). However, there was significant heterogeneity across the observational studies. By sensitivity analysis of the association between MACEs and use of an individual PPI after pooling studies reporting only the HR or the incidences of MACEs at a 1-year endpoint, the direction of estimates remained unchanged (Supplementary Table S5).

### Myocardial Infarction

Three RCTs, which included 5,856 patients (Bhatt et al., 2010; Jensen et al., 2017; Zhang et al., 2019), reported no significant difference in the incidence of MI between the experimental and control groups (RR = 0.93 [95% CI = 0.76–1.15], *p* = 0.50, I^2^ = 0%; Figure 2). Of the observational studies, post-hoc analysis of RCTs conducted by O’Donoghue et al. (2009) found no significant interactions among different types of PPIs with the use of clopidogrel or prasugrel, or an increased risk of MI. Moreover, post-hoc analysis of a RCT conducted by Goodman et al. (2012) reported no association between the use of PPIs and the risk of MI during ticagrelor treatment. Meanwhile, a case-control study by Juurlink et al. (2009) found no difference in the incidence of MI between patient groups treated with or without pantoprazole added to a clopidogrel-based regimen. Given the sparsity of data, meta-analysis of the observational studies for the incidence of MI was not performed. However, there was no trend toward an increase in the incidence of MI in the groups treated with PPIs.

### All-Cause Mortality

**FIGURE 4:**
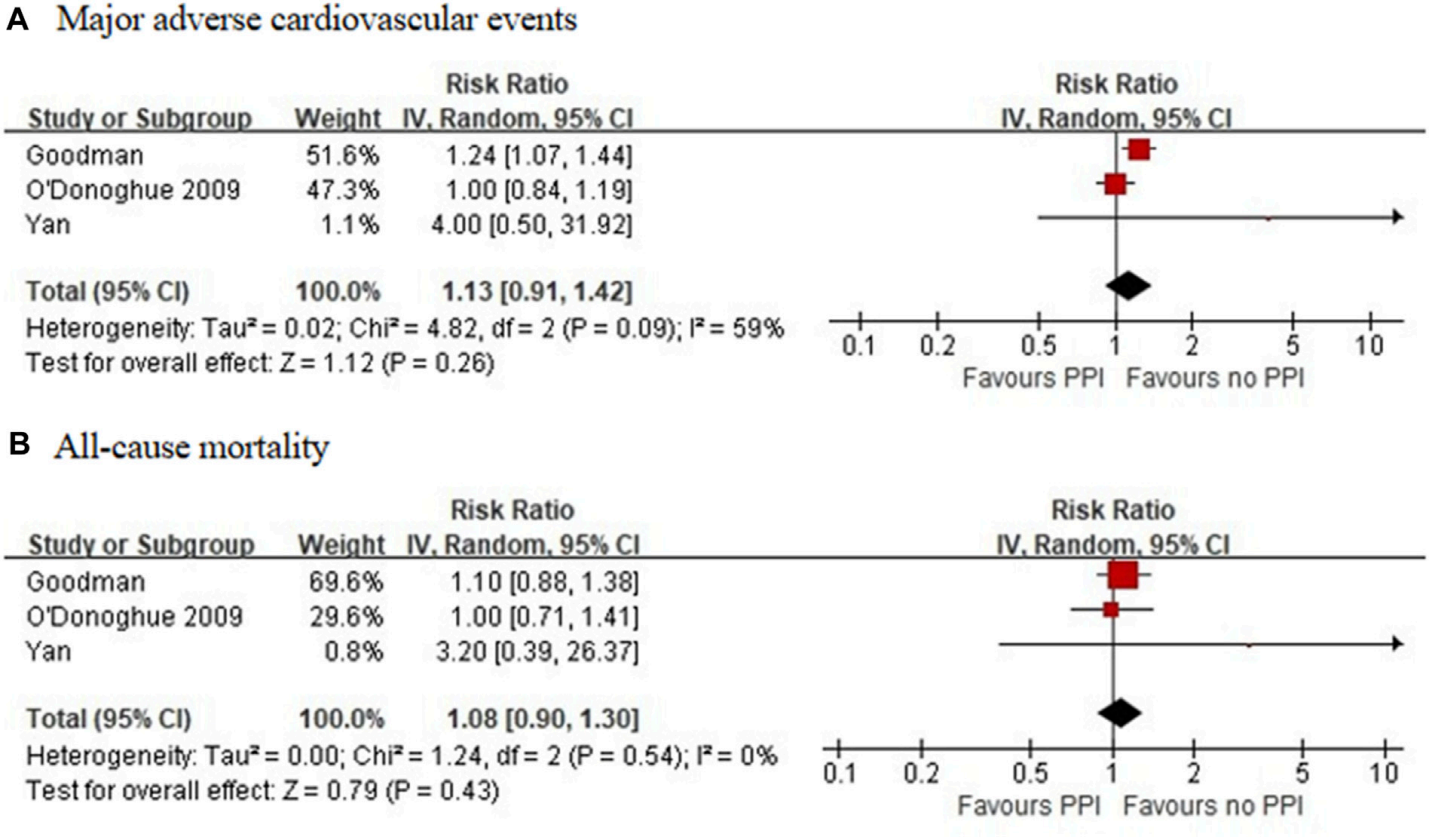
Meta-analysis of observational studies of major adverse cardiovascular events **(A)** and all-cause mortality **(B)** with other P2Y12 inhibitors and proton pump inhibitor use.

Four RCTs, which included 6,672 patients (Gao et al., 2009; Bhatt et al., 2010; Wu et al., 2011; Jensen et al., 2017), reported no significant differences in the incidence of all-cause mortality between the experimental and control groups (RR = 0.79 [95% CI = 0.50–1.23], *p* = 0.30, I^2^ = 23%; Figure 2). Of the 16 observational studies, none reported the risk of all-cause mortality with the use of an individual PPI combined with clopidogrel. A meta-analysis of three observational studies revealed no increased risk of all-cause mortality associated with PPIs as a class co-administered with prasugrel or ticagrelor (RR = 1.08 [95% CI, 0.90-1.30], *p* = 0.43, I^2^ = 0%; Figure 4) (O'Donoghue et al., 2009; Goodman et al., 2012; Yan et al., 2016).

### Gastrointestinal Bleeding

**FIGURE 5:**
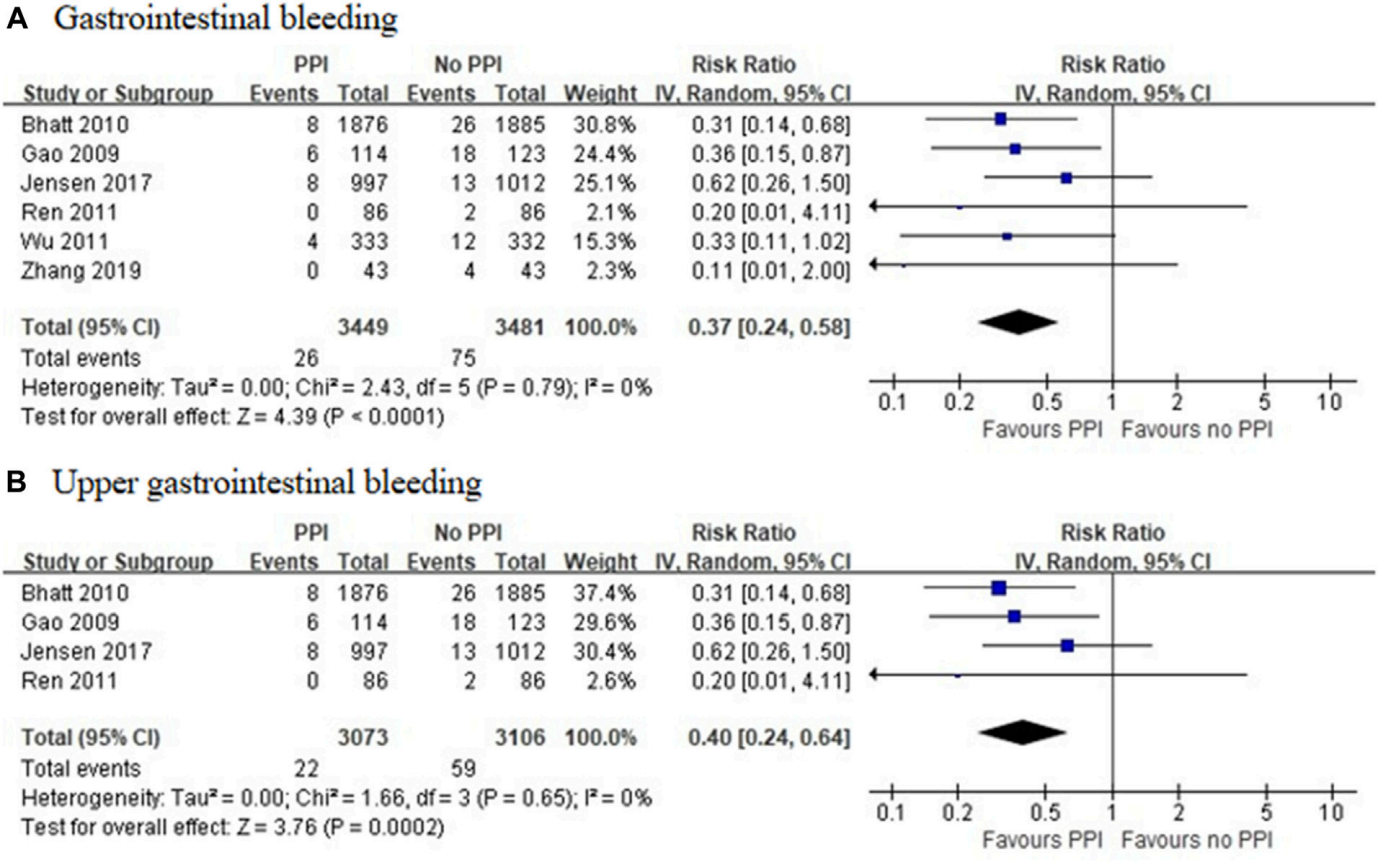
Meta-analysis of randomised controlled trials of gastrointestinal bleeding **(A)** and upper gastrointestinal bleeding **(B)** with dual antiplatelet therapy and proton pump inhibitor use.

**FIGURE 6:**
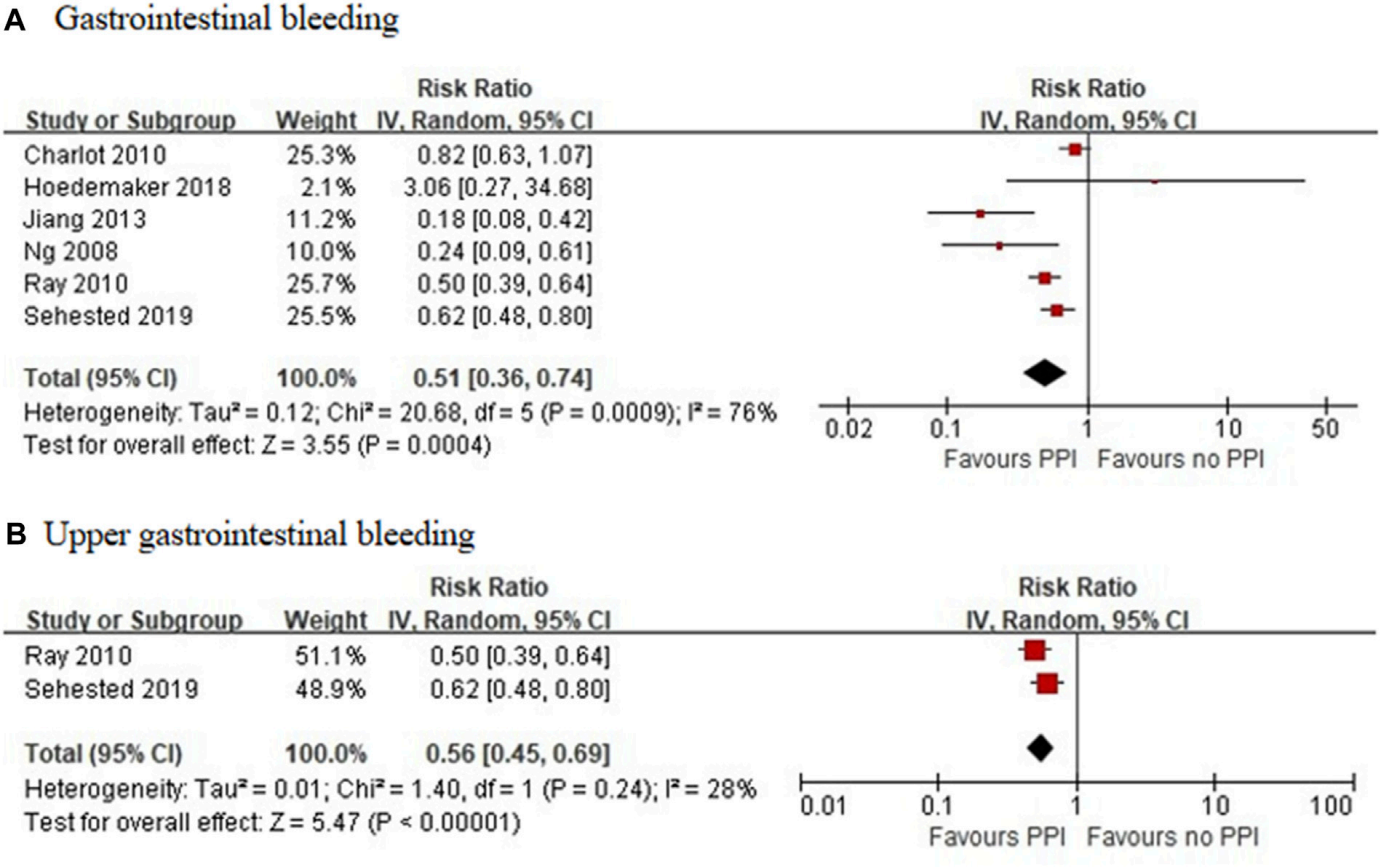
Meta-analysis of observational studies of gastrointestinal bleeding **(A)** and upper gastrointestinal bleeding **(B)** with dual antiplatelet therapy and proton pump inhibitor use.

Six RCTs, which included 6,930 patients (Gao et al., 2009; Bhatt et al., 2010; Ren et al., 2011; Wu et al., 2011; Jensen et al., 2017; Zhang et al., 2019), reported a significant reduction in the incidence of GI bleeding in the PPI group (RR = 0.37 [95% CI = 0.24–0.58], *p* < 0.001, I^2^ = 0%). Studies that specified data on UGI bleeding found that the rate of UGI bleeding was also reduced in the PPI group as compared with the non-PPI group (RR = 0.40 [95% CI = 0.24–0.64], *p* < 0.001, I^2^ = 0%; Figure 5) (Gao et al., 2009; Bhatt et al., 2010; Ren et al., 2011; Jensen et al., 2017). Six observational studies (Ng et al., 2008; Charlot et al., 2010; Ray et al., 2010; Jiang et al., 2013; Hoedemaker et al., 2019; Sehested et al., 2019) reported that the concomitant use of PPIs plus DAPT was associated with a decreased risk of GI bleeding (RR = 0.51 [95% CI = 0.36–0.74], *p* < 0.001, I^2^ = 76%). This interaction also existed with a significant reduction in heterogeneity, when specific data on UGI bleeding were pooled (RR = 0.56 [95% CI = 0.45–0.69], *p* < 0.001, I^2^ = 28%; Figure 6) (Ray et al., 2010; Sehested et al., 2019).

## Discussion

Although most previous studies mainly focused on the effects of PPIs on CV outcomes in patients receiving clopidogrel (Kwok and Loke, 2010; Siller-Matula et al., 2010; Chen et al., 2012; Gerson et al., 2012; Kwok et al., 2013; Melloni et al., 2015; Sherwood et al., 2015; Serbin et al., 2016; Bundhun et al., 2017; Niu et al., 2017; Demcsák et al., 2018; Khan et al., 2018), the current study is the first comprehensive systematic review to examine the efficacy and safety profile of PPIs combined with DAPT in patients after PCI or ACS and is, to the best of our knowledge, the only study to evaluate the safety of PPIs with the concomitant use of other P2Y_12_ inhibitors.

This review of 22 studies with 6,930 patients from RCTs to 183,546 patients from observational studies showed that the use of PPIs probably reduced the risk of GI bleeding and UGI bleeding relative to no PPI use. A meta-analysis of RCTs to assess the effect of PPIs as a class suggested that the use of PPIs was not associated with increased risks of MACEs, MI, or all-cause mortality when co-administered with DAPT. However, we found differences in the risks of MACEs between PPI subtypes concomitant with the use of clopidogrel and there was no increased risk of MACEs and all-cause mortality when PPIs (as a class) were used with other P2Y_12_ inhibitors.

Post-discharge bleeding after ACS is associated with a similar increase in subsequent all-cause mortality in patients treated with or without PCI and has an equivalent prognostic impact as that of post-discharge MI (Marquis-Gravel et al., 2020). Therefore, PPIs are frequently co-administered with DAPT to reduce the risk of GI bleeding. However, several studies have suggested that the antiplatelet effects of clopidogrel could be attenuated by PPIs (Gilard et al., 2006; O'Donoghue et al., 2009; Disney et al., 2011) because PPIs may competitively inhibit cytochrome P450 2C19 (CYP2C19), which is involved in the metabolic activation of clopidogrel, leading to interference with the metabolism of clopidogrel into active metabolites. Of all PPIs, omeprazole and esomeprazole have high potency inhibitory effects against clopidogrel, while pantoprazole seems confer a lesser effect. In addition, the acidic environment of the GI tract is conducive to drug absorption, and therefore the use of a PPI could diminish or slow drug absorption.

Our findings from the pooled analysis of RCTs show that the use of PPIs was not associated with the risks of adverse CV outcomes and data from observational studies showed that omeprazole, esomeprazole, and rabeprazole were not associated with MACEs. These findings are consistent with a previous meta-analysis of RCTs, which found no association or increased risks of adverse CV outcomes with the use of PPIs (Khan et al., 2018). A post-analysis of RCTs conducted by O’Donoghue et al. (2009) reported that the mean inhibition of platelet aggregation was significantly lower for the PPI group as compared with the non-PPI group, despite the use of higher loading and maintenance doses of clopidogrel. However, there was no association between PPI use and adverse CV outcomes after 15 months of follow-up. The study conducted by Zhu et al. (2017) noted similar results, that although inhibition of platelet aggregation was significantly lower in the PPI group than in the non-PPI group, concomitant use of a PPI was not associated with increased risks of MACEs or cerebrovascular events. These findings partly indicate that a modest attenuation of the antiplatelet effects of clopidogrel is insufficient to have an impact on clinical outcomes. These results are similar to those of a previous study of statin-clopidogrel interactions, which reported that atorvastatin induced attenuation of the antiplatelet effects of clopidogrel in a dose-dependent manner, but had no effect on clinical outcomes (Saw et al., 2003; Gorchakova et al., 2004; Steinhubl and Akers, 2006).

Analysis of observational studies found an inconsistent association between the use of each PPI subtype and the increased risks of MACEs. Importantly, we found that pantoprazole, which conveys relatively lower inhibitory potency against CYP2C19, as compared with other PPIs, was associated with increased risks of MACEs. As in observational studies, the use of a PPI was not randomized or at the discretion of physician, while there was a general trend observed that patients using PPIs were older with more comorbidities and used more co-medications. Although all of the included observational studies in this meta-analysis were adjusted for potential confounders, the unmeasured risk factors and potential residual confounding factors of imperfectly measured variables might also have influenced the results. For example, a prospective cohort study of 97,503 participants with a relatively long-term follow-up period found that PPI use was associated with an increased risk of ischemic stroke after controlling for established risk factors of stroke (Nguyen et al., 2018). However, this association was substantially reduced after additional adjustment for potential indications for PPI use, suggestive of significant confounding, but no causal relationship. Furthermore, the genetic polymorphism of CYP2C19 is yet another potential confounding factor. Previous studies found that patients with loss-of-function of CYP2C19 alleles were at a significantly higher risk for adverse CV outcomes (Hulot et al., 2010; Goodman et al., 2012), suggesting that these patients are more vulnerable to further attenuation of the antiplatelet effects of clopidogrel by PPIs. However, the evidence of adverse CV outcomes in clopidogrel-treated patients with CYP2C19 loss-of-function alleles remains controversial.

The present systematic review also assessed the safety of PPIs independent of clopidogrel use and found that the use of PPIs was not associated with increased risks of MACEs or all-cause mortality. This finding is significant because previous studies have questioned whether there exists a potential association between PPI use and adverse ischemic events directly conferred by PPIs (Shah et al., 2015; Wang et al., 2017; Li et al., 2019). To date, several pathophysiological hypotheses have been proposed (Wilhelm et al., 2013). Asymmetric dimethylarginine (ADMA), an endogenous and competitive inhibitor of endothelial nitric oxide synthase, has been thought to be associated with an increased risk of CV disease. Human dimethylarginine dimethylaminohydrolase (DDAH) is mainly responsible for the metabolism of 80% of ADMA, while PPIs can directly inhibit DDAH activity, thus, increasing ADMA levels and decreasing the release of nitric oxide, leading to the disruption of vascular homeostasis (Ghebremariam et al., 2013). In addition, PPIs can attenuate vitamin C uptake and increase the activation of reactive oxygen species-dependent pathways, while subsequently inhibiting DDAH activity (Chen et al., 2019). PPIs can also increase plasma ADMA levels and impair endothelium-dependent vasodilation through elevations in plasma homocysteine levels by interfering with the absorption of vitamin B12, which is responsible for the conversion of homocysteine to cysteine (Wilhelm et al., 2013). By contrast, data from another study found no association between PPI use and increased plasma ADMA levels in coronary artery disease (Kruszelnicka et al., 2016).

Another mechanism may be related to the senescence of human endothelial cells (ECs) induced by long-term exposure to PPIs. PPIs can impair lysosomal acidification and subsequent proteostasis, thereby promoting the aging of ECs. Besides, chronic exposure to PPIs upregulates the expression levels of genes associated with endothelial-to-mesenchymal transition (EndoMT) and has been correlated with histological changes consistent with EndoMT. EndoMT is a marker of aging ECs, which may play an important role in CV disease (Yepuri et al., 2016). In addition, Costarelli et al. (2017) investigated changes in gene expression patterns occurring in senescent and non-senescent human coronary artery endothelial cells (HCAECs) following long-term use of high-dose PPIs, and found that PPIs induced down-regulation of anti-atherogenic chemokines (CXCL11, CXCL12 and CX3CL1) in senescent cells, suggesting that PPIs can activate pro-atherogenic pathways, which increases the risk of CV disease in older patients by changing the secretory phenotype of senescent HCAECs.

Current evidence also suggests that PPI-induced hypomagnesemia may mediate adverse CV effects. Magnesium plays a key role in maintaining cardiovascular homeostasis. PPI-induced hypomagnesemia, which accounts for only 1.0% of all reported PPI-related adverse effects (Luk et al., 2013), may cause polymorphic ventricular tachycardia, cardiac conduction disturbances, and even sudden death (Chrysant, 2019). Although the exact mechanism underlying PPI-induced hypomagnesemia remains unclear, it may be related to defective absorption of magnesium through active or passive transport processes, or excessive loss into the intestinal lumen (Cundy and Mackay, 2011). Given the low incidence of PPI-induced hypomagnesemia, physicians should be aware that hypomagnesemia may also be caused by other drugs, such as diuretics and digoxin.

Other related mechanisms that increase the risk of CV events after long-term PPI administration include the influence of PPIs on the gut microbiota and the PPI-induced increase in chromogranin-A (CgA) release. Long-term use of PPIs decreases gastric acid barrier function, leading to the invasion and colonization of exogenous pathogenic bacteria in the intestine, resulting in gut microbiota imbalance and an increased risk of atherosclerosis (Manolis et al., 2020). High CgA plasma levels are associated with an increased risk of mortality after MI or ACS, as well as heart failure due to the increased release of endothelin-1 from ECs (D’amico et al., 2014), as endothelin-1 has been implicated in CVD and vascular dysfunction via the promotion of inflammation and atherosclerosis (Böhm and Pernow, 2007).

However, there is still no solid evidence to demonstrate whether these potential mechanisms will translate into an increased risk of adverse clinical outcomes. Post-hoc analysis of the PLATO trial conducted by Goodman et al. (2012) showed that PPIs may adversely affect CV outcomes in patients with ACS when co-administered with ticagrelor, which does not require metabolism by CYP2C19, indicating the possible involvement of other mechanisms. However, before randomization, they also found that PPI use was associated with greater risks of adverse CV outcomes, as compared with landmark analyses at days 2, 4, 9, 30, 60, 90, and 180 after randomization in both the ticagrelor and clopidogrel groups. This study concluded that PPI use was a marker of a greater risk of adverse clinical outcomes. A meta-analysis conducted by Batchelor et al. (2018) found that PPI monotherapy was associated with an increased risk of adverse CV outcomes using pooled data from observational studies, but not from RCTs. Therefore, the most plausible explanation for these results was that the increased risk of adverse CV outcomes associated with PPI use may be due to the characteristics of the patients using these drugs. Thus, it is important to note that such an association may not necessarily reflect a direct drug effect.

Finally, although potential unmeasured bias and confounding factors are better controlled in RCTs than non-randomized studies, the RCTs included in the present analysis included smaller numbers of patients and tended to have shorter follow-up periods as compared with the observational studies. Furthermore, it remains unclear whether the use of PPIs is associated with an increased risk for dose-dependent adverse CV outcomes. Sehested et al. (2018) suggested that, as compared with low-dose therapy, high-dose PPI therapy was associated with a greater risk for adverse CV outcomes. In contrast, Ray et al. (2010) indicated that concurrent use of clopidogrel and high-dose PPI therapy was not associated with an increased risk for adverse CV outcomes. Hence, further RCTs with long-term follow-ups are needed to further elucidate the potential risk of high-dose and long-term exposure to PPIs. On the other hand, switching PPIs for other drugs to control GI bleeding may be a viable alternative approach for patients requiring long-term gastroprotection.

### Limitations

As a limitation to statistical validity, data were pooled from studies reporting different measures of association. However, given the overall low incidence of GI bleeding, the HRs are assumed to approximate the RRs, which was unlikely to have a significant impact on the meta-analysis results. Due to the higher rates of MACEs, sensitivity analysis was performed of studies reporting HRs only, and the direction of the estimates remained unchanged. Moreover, the effect size was not dependent on how the measure of effect was expressed. The pooling of different measures of effect size has also been adopted in other studies under similar circumstances (Siller-Matula et al., 2010; Kwok et al., 2011; Lambert et al., 2015; Sherwood et al., 2015; Batchelor et al., 2018). Furthermore, the included observational studies retrieved data from prescription and pharmacy dispensing record databases to ascertain exposure. Since PPIs could have been either initiated or discontinued throughout the follow-up period, actual adherence could not be assessed for individual patients. Finally, differences in the end point definitions may have weakened the results of quantitative analysis.

## Conclusion

The current systematic review and meta-analysis found the use of PPIs significantly reduced the risk of GI bleeding in patients treated with DAPT and there was no clear evidence of an association between PPI use and adverse CV outcomes. Due to the limitations of both RCTs and observational studies, further RCTs with long-term follow-up periods are needed to further evaluate the safety of PPIs with concomitant DAPT use in patients after ACS or PCI.

## Data Availability

The original contributions presented in the study are included in the article/Supplementary Material, further inquiries can be directed to the corresponding author.
