# Supplementary material for: Clinical Outcomes of Concomitant Use of Proton Pump Inhibitors and Dual Antiplatelet Therapy: A Systematic Review and Meta-Analysis
Source: Front Pharmacol. 2021 Aug 2;12:694698. doi: 10.3389/fphar.2021.694698 (PMC8366318; doi:10.3389/fphar.2021.694698)
Supplement: Supplementary file 2 [file Table2.DOCX]

**Supplement 2. Search strategies**

**PUBMED**

1 ("Dual Anti-Platelet Therapy"[Mesh]) OR ((((((("Dual Anti-Platelet Therapy"[Title/Abstract]) OR "Dual Antiplatelet Therapy"[Title/Abstract]) OR "Anti-Platelet Therapy, Dual"[Title/Abstract]) OR "Dual Anti-Platelet Therapies"[Title/Abstract]) OR "Dual Anti Platelet Therapy"[Title/Abstract]) OR "Anti-Platelet Therapies, Dual"[Title/Abstract]) OR "DAPT"[Title/Abstract])

2 ("Clopidogrel"[Mesh]) OR ((((((((((((((((("Clopidogrel"[Title/Abstract]) OR "SC 25989C"[Title/Abstract]) OR "SC 25990C"[Title/Abstract]) OR "SR 25989"[Title/Abstract]) OR "Clopidogrel-Mepha"[Title/Abstract]) OR "Clopidogrel Mepha"[Title/Abstract]) OR "Clopidogrel Sandoz"[Title/Abstract]) OR "Iscover"[Title/Abstract]) OR "Clopidogrel Napadisilate"[Title/Abstract]) OR "Clopidogrel Hydrochloride"[Title/Abstract]) OR "PCR 4099"[Title/Abstract]) OR "PCR-4099"[Title/Abstract]) OR "Clopidogrel Besylate"[Title/Abstract]) OR "Clopidogrel Besilate"[Title/Abstract]) OR "Clopidogrel, (+)(S)-isomer"[Title/Abstract]) OR "Plavix"[Title/Abstract]) OR "Clopidogrel Bisulfate"[Title/Abstract])

3 ("Ticagrelor"[Mesh]) OR ((((((("Ticagrelor"[Title/Abstract]) OR "Brilique"[Title/Abstract]) OR "AZD 6140"[Title/Abstract]) OR "AZD6140"[Title/Abstract]) OR "AZD-6140"[Title/Abstract]) OR "Brilinta"[Title/Abstract]) OR "3-(7-((2-(3,4-Difluorophenyl)cyclopropyl)amino)-5-(propylthio)-3H-(1-3)-triazolo(4,5-d)pyrimidin-3-yl)-5-(2-hydroxyethoxy)cyclopentane-1,2-diol"[Title/Abstract])

4 ("Prasugrel Hydrochloride"[Mesh]) OR ((((((((((((((("Prasugrel Hydrochloride"[Title/Abstract]) OR "Hydrochloride, Prasugrel"[Title/Abstract]) OR "Prasugrel HCl"[Title/Abstract]) OR "HCl, Prasugrel"[Title/Abstract]) OR "CS 747"[Title/Abstract]) OR "747, CS"[Title/Abstract]) OR "CS-747"[Title/Abstract]) OR "CS747"[Title/Abstract]) OR "Prasugrel"[Title/Abstract]) OR "Efient"[Title/Abstract]) OR "Effient"[Title/Abstract]) OR "LY 640315"[Title/Abstract]) OR "640315, LY"[Title/Abstract]) OR "LY640315"[Title/Abstract]) OR "LY-640315"[Title/Abstract])

5 (((((((("P2Y12 antagonist"[Title/Abstract]) OR "P2Y12 antagonists"[Title/Abstract]) OR "P2Y12 inhibitor"[Title/Abstract]) OR "P2Y12 inhibitors"[Title/Abstract]) OR "P2Y12 receptor antagonist"[Title/Abstract]) OR "P2Y12 receptor antagonists"[Title/Abstract]) OR "P2Y12 receptor inhibitor"[Title/Abstract]) OR "P2Y12 receptor inhibitors"[Title/Abstract]) OR "P2Y12 Inhibition"[Title/Abstract]

6 ("Percutaneous Coronary Intervention"[Mesh]) OR ((((((((((((("Percutaneous Coronary Intervention"[Title/Abstract]) OR "Coronary Intervention, Percutaneous"[Title/Abstract]) OR "Coronary Interventions, Percutaneous"[Title/Abstract]) OR "Intervention, Percutaneous Coronary"[Title/Abstract]) OR "Interventions, Percutaneous Coronary"[Title/Abstract]) OR "Percutaneous Coronary Interventions"[Title/Abstract]) OR "Percutaneous Coronary Revascularization"[Title/Abstract]) OR "Coronary Revascularization, Percutaneous"[Title/Abstract]) OR "Coronary Revascularizations, Percutaneous"[Title/Abstract]) OR "Percutaneous Coronary Revascularizations"[Title/Abstract]) OR "Revascularization, Percutaneous Coronary"[Title/Abstract]) OR "Revascularizations, Percutaneous Coronary"[Title/Abstract]) OR "PCI"[Title/Abstract])

7 ("Acute Coronary Syndrome"[Mesh]) OR ((((((("Acute Coronary Syndrome"[Title/Abstract]) OR "Acute Coronary Syndromes"[Title/Abstract]) OR "Coronary Syndrome, Acute"[Title/Abstract]) OR "Coronary Syndromes, Acute"[Title/Abstract]) OR "Syndrome, Acute Coronary"[Title/Abstract]) OR "Syndromes, Acute Coronary"[Title/Abstract]) OR "ACS"[Title/Abstract])

8 1 or 2 or 3 or 4 or 5 or 6 or 7

9 ("Proton Pump Inhibitors"[Mesh]) OR (((("Proton Pump Inhibitors"[Title/Abstract]) OR "Proton Pump Inhibitor"[Title/Abstract]) OR "Inhibitors, Proton Pump"[Title/Abstract]) OR "PPI"[Title/Abstract])

10 ("Omeprazole"[Mesh]) OR ((((((((("Omeprazole"[Title/Abstract]) OR "Prilosec"[Title/Abstract]) OR "Omeprazole Sodium"[Title/Abstract]) OR "Sodium, Omeprazole"[Title/Abstract]) OR "H 168-68"[Title/Abstract]) OR "H 168 68"[Title/Abstract]) OR "H 16868"[Title/Abstract]) OR "Omeprazole Magnesium"[Title/Abstract]) OR "Magnesium, Omeprazole"[Title/Abstract])

11 ("Esomeprazole"[Mesh]) OR ((((((((Esomeprazole[Title/Abstract]) OR "Esomeprazole Strontium"[Title/Abstract]) OR "Strontium, Esomeprazole"[Title/Abstract]) OR "Esomeprazole Magnesium"[Title/Abstract]) OR "Nexium"[Title/Abstract]) OR "Esomeprazole Potassium"[Title/Abstract]) OR "Esomeprazole Strontium Anhydrous"[Title/Abstract]) OR "Esomeprazole Sodium"[Title/Abstract])

12 ("Pantoprazole"[Mesh]) OR (((((((((((("Pantoprazole"[Title/Abstract]) OR "Pantoprazole Sodium"[Title/Abstract]) OR ("SK[Title/Abstract] AND F 96022"[Title/Abstract])) OR "SKF-96022"[Title/Abstract]) OR "SKF 96022"[Title/Abstract]) OR "SKF96022"[Title/Abstract]) OR ("SK[Title/Abstract] AND F-96022"[Title/Abstract])) OR ("SK[Title/Abstract] AND F96022"[Title/Abstract])) OR "BY 1023"[Title/Abstract]) OR "BY-1023"[Title/Abstract]) OR "BY1023"[Title/Abstract]) OR "Protonix"[Title/Abstract])

13 ("Rabeprazole"[Mesh]) OR ((((((((((((("Rabeprazole"[Title/Abstract]) OR "2-((4-(3-methoxypropoxy)-3-methylpyridin-2-yl)methylsulfinyl)-1H-benzimidazole"[Title/Abstract]) OR "Dexrabeprazole"[Title/Abstract]) OR "E 3810"[Title/Abstract]) OR "E3810"[Title/Abstract]) OR "Pariet"[Title/Abstract]) OR "Rabeprazole Sodium"[Title/Abstract]) OR "Sodium, Rabeprazole"[Title/Abstract]) OR "1H-Benzimidazole, 2-(((4-(3-methoxypropoxy)-3-methyl-2-pyridinyl)methyl)sulfinyl)-, Sodium Salt"[Title/Abstract]) OR "Aciphex"[Title/Abstract]) OR "LY-307640"[Title/Abstract]) OR "LY 307640"[Title/Abstract]) OR "LY307640"[Title/Abstract])

14 ("Lansoprazole"[Mesh]) OR (((((((((((((((((((((((("Lansoprazole"[Title/Abstract]) OR "Lansoprazol"[Title/Abstract]) OR "2-(((3-Methyl-4-(2,2,2-trifluoroethoxy)-2-pyridyl)methyl)sulfinyl)benzimidazole"[Title/Abstract]) OR "Lansoprazoles"[Title/Abstract]) OR "Ogastro"[Title/Abstract]) OR "AG 1749"[Title/Abstract]) OR "AG-1749"[Title/Abstract]) OR "AG1749"[Title/Abstract]) OR "Agopton"[Title/Abstract]) OR "Bamalite"[Title/Abstract]) OR "Lansol"[Title/Abstract]) OR "Lansoprazole Sodium"[Title/Abstract]) OR "Sodium, Lansoprazole"[Title/Abstract]) OR "Lanzor"[Title/Abstract]) OR "Monolitum"[Title/Abstract]) OR "Opiren"[Title/Abstract]) OR "Prevacid"[Title/Abstract]) OR "Pro Ulco"[Title/Abstract]) OR "Promeco"[Title/Abstract]) OR "Takepron"[Title/Abstract]) OR "Ulpax"[Title/Abstract]) OR "Zoton"[Title/Abstract]) OR "Ogast"[Title/Abstract]) OR "Prezal"[Title/Abstract])

15 ("Dexlansoprazole"[Mesh]) OR (((((((((((((((((("Dexlansoprazole"[Title/Abstract]) OR "Lansoprazole, R-Isomer"[Title/Abstract]) OR "Lansoprazole, R Isomer"[Title/Abstract]) OR "R-Isomer Lansoprazole"[Title/Abstract]) OR "2-((R)-((3-Methyl-4-(2,2,2-trifluoroethoxy)-2-pyridinyl)methyl)sulfinyl)-1H-benzimidazole"[Title/Abstract]) OR "R-Lansoprazole"[Title/Abstract]) OR "R Lansoprazole"[Title/Abstract]) OR "Dexlansoprazole Sesquihydrate"[Title/Abstract]) OR "TAK 390MR"[Title/Abstract]) OR "TAK390MR"[Title/Abstract]) OR "TAK-390MR"[Title/Abstract]) OR "TAK-390"[Title/Abstract]) OR "TAK 390"[Title/Abstract]) OR "TAK390"[Title/Abstract]) OR "Dexilant"[Title/Abstract]) OR "T-168390"[Title/Abstract]) OR "T 168390"[Title/Abstract]) OR "T168390"[Title/Abstract])

16 ((("ilaprazole"[Title/Abstract]) OR "IY 81149"[Title/Abstract]) OR "IY81149"[Title/Abstract]) OR "IY-81149"[Title/Abstract]

17 ((("leminoprazole"[Title/Abstract]) OR "(+-)-2-((2-(isobutylmethylamino)benzyl)sulfinyl)-1H-benzimidazole"[Title/Abstract]) OR "NC 1300-O-3"[Title/Abstract]) OR "NC-1300-O-3"[Title/Abstract]

18 (("picoprazole"[Title/Abstract]) OR "2-((2-(3-methyl)pyridyl-methyl)sulfinyl)-5-methoxycarbonyl-6-methylbenzimidazole"[Title/Abstract]) OR "H 149-94"[Title/Abstract]

19 pumaprazole[Title/Abstract]

20 ("timoprazole"[Title/Abstract]) OR "2-((2-pyridylmethyl)sulfinyl)benzimidazole"[Title/Abstract]

21 saviprazole[Title/Abstract]

22 ((((("tenatoprazole"[Title/Abstract]) OR "5-methyl-2((4-methoxy-3,5-dimethyl-2-pyridyl)methyl)sulfinyl-imidazo(4,5-b)pyridine"[Title/Abstract]) OR "5-methoxy-2-(((4-methoxy-3,5-dimethylpyrid-2-yl)methyl)sulfinyl)-1H-imidazo(4,5-b)pyridine"[Title/Abstract]) OR "TU 199"[Title/Abstract]) OR "TU199"[Title/Abstract]) OR "TU-199"[Title/Abstract]

23 9 or 10 or 11 or 12 or 13 or 14 or 15 or 16 or 17 or 18 or 19 or 20 or 21 or 22

24 8 and 23

**COCHRANE LIBRARY**

#1 MeSH descriptor: [Dual Anti-Platelet Therapy] explode all trees

#2 ((Dual Anti-Platelet Therapy) OR (Dual AntiPlatelet Therapy) OR (Anti-Platelet Therapy, Dual) OR (Dual Anti-Platelet Therapies) OR (Anti-Platelet Therapies, Dual) OR (Dual Anti Platelet Therapy)):ti,ab,kw

#3 #1 OR #2

#4 MeSH descriptor: [Clopidogrel] explode all trees

#5 (Clopidogrel OR (Clopidogrel Bisulfate) OR Plavix OR (Clopidogrel Besylate) OR (Clopidogrel Besilate) OR Iscover OR (Clopidogrel Sandoz) OR (Clopidogrel Hydrochloride) OR (SC 25990C) OR (Clopidogrel Napadisilate) OR (Clopidogrel Mepha) OR (Clopidogrel-Mepha) OR (SR 25989) OR (SC 25989C) OR (PCR 4099) OR (PCR-4099)):ti,ab,kw

#6 #4 OR #5

#7 MeSH descriptor: [Ticagrelor] explode all trees

#8 (Ticagrelor OR (AZD6140) OR (AZD 6140) OR (AZD-6140) OR Brilinta OR Brilique):ti,ab,kw

#9 #7 OR #8

#10 MeSH descriptor: [Prasugrel Hydrochloride] explode all trees

#11 ((Prasugrel Hydrochloride) OR (LY 640315) OR (LY640315) OR (640315, LY) OR (LY-640315) OR (CS-747) OR (CS747) OR (747, CS) OR (CS 747) OR Efient OR Effient OR (Prasugrel HCl) OR (HCl, Prasugrel) OR (Hydrochloride, Prasugrel) OR Prasugrel):ti,ab,kw

#12 #10 OR #11

#13 ((P2Y12 antagonist) OR (P2Y12 antagonists) OR (P2Y12 inhibitor) OR (P2Y12 inhibitors) OR (P2Y12 receptor antagonist) OR (P2Y12 receptor antagonists) OR (P2Y12 receptor inhibitor) OR (P2Y12 receptor inhibitors) OR (P2Y12 Inhibition)):ti,ab,kw

#14 MeSH descriptor: [Percutaneous Coronary Intervention] explode all trees

#15 ((Percutaneous Coronary Intervention) OR (Revascularizations, Percutaneous Coronary) OR (Percutaneous Coronary Revascularization) OR (Coronary Interventions, Percutaneous) OR (Coronary Revascularization, Percutaneous) OR (Coronary Intervention, Percutaneous) OR (Intervention, Percutaneous Coronary) OR (Percutaneous Coronary Revascularizations) OR (Coronary Revascularizations, Percutaneous) OR (Percutaneous Coronary Interventions) OR (Revascularization, Percutaneous Coronary) OR (Interventions, Percutaneous Coronary)):ti,ab,kw

#16 #14 OR #15

#17 MeSH descriptor: [Acute Coronary Syndrome] explode all trees

#18 ((Acute Coronary Syndrome) OR (Acute Coronary Syndromes) OR (Syndrome, Acute Coronary) OR (Coronary Syndromes, Acute) OR (Syndromes, Acute Coronary) OR (Coronary Syndrome, Acute)):ti,ab,kw

#19 #17 OR #18

#20 (DAPT):ti,ab,kw

#21 (ACS):ti,ab,kw

#22 (PCI):ti,ab,kw

#23 #3 OR #6 OR #9 OR #12 OR #13 OR #16 OR #19 OR #20 OR #21 OR #22

#24 MeSH descriptor: [Proton Pump Inhibitors] explode all trees

#25 ((Proton Pump Inhibitors) OR (Proton Pump Inhibitor) OR (Inhibitors, Proton Pump)):ti,ab,kw

#26 #24 OR #25

#27 (PPI):ti,ab,kw

#28 MeSH descriptor: [Omeprazole] explode all trees

#29 (Omeprazole OR (H 168 68) OR (H 16868) OR (Omeprazole Sodium) OR (Sodium, Omeprazole) OR Prilosec OR (Omeprazole Magnesium) OR (Magnesium, Omeprazole)):ti,ab,kw

#30 #28 OR #29

#31 MeSH descriptor: [Esomeprazole] explode all trees

#32 (Esomeprazole OR (Esomeprazole Strontium) OR (Strontium, Esomeprazole) OR (Esomeprazole Strontium Anhydrous) OR (Esomeprazole Sodium) OR Nexium OR (Esomeprazole Potassium) OR (Esomeprazole Magnesium)):ti,ab,kw

#33 #31 OR #32

#34 MeSH descriptor: [Pantoprazole] explode all trees

#35 (Pantoprazole OR SKF96022 OR SKF-96022 OR SK&F-96022 OR (SK&F 96022) OR (SKF 96022) OR SK&F96022 OR Protonix OR BY1023 OR BY-1023 OR (BY 1023) OR (Pantoprazole Sodium)):ti,ab,kw

#36 #34 OR #35

#37 MeSH descriptor: [Rabeprazole] explode all trees

#38 (Rabeprazole OR Aciphex OR E3810 OR (E 3810) OR Pariet OR Dexrabeprazole OR (Rabeprazole Sodium) OR (Sodium, Rabeprazole) OR (LY 307640) OR LY307640 OR LY-307640):ti,ab,kw (Word variations have been searched)

#39 #37 OR #38

#40 MeSH descriptor: [Lansoprazole] explode all trees

#41 (Lansoprazole OR Lansoprazoles OR Lansoprazol OR Opiren OR Ulpax OR Agopton OR (AG 1749) OR AG-1749 OR AG1749 OR Ogastro OR Lansol OR Ogast OR Zoton OR Takepron OR Lanzor OR Promeco OR Prevacid OR (Pro Ulco) OR Monolitum OR Prezal OR Bamalite OR (Sodium, Lansoprazole) OR (Lansoprazole Sodium)):ti,ab,kw

#42 #40 OR #41

#43 MeSH descriptor: [Dexlansoprazole] explode all trees

#44 (Dexlansoprazole OR Dexilant OR TAK-390MR OR (TAK 390MR) OR TAK390MR OR (Dexlansoprazole Sesquihydrate) OR (TAK 390) OR TAK-390 OR TAK390 OR (T 168390) OR T168390 OR T-168390 OR (R Lansoprazole) OR (Lansoprazole, R-Isomer) OR (R-Isomer Lansoprazole) OR (R-Lansoprazole) OR (Lansoprazole, R Isomer)):ti,ab,kw

#45 #43 OR #44

#46 (ilaprazole OR (IY 81149) OR IY81149 OR IY-81149):ti,ab,kw

#47 (leminoprazole):ti,ab,kw

#48 (picoprazole):ti,ab,kw

#49 (pumaprazole):ti,ab,kw

#50 (timoprazole):ti,ab,kw

#51 (saviprazole):ti,ab,kw

#52 (tenatoprazole OR (TU 199) OR TU199 OR TU-199):ti,ab,kw

#53

#26 OR #27 OR #30 OR #33 OR #36 OR #39 OR #42 OR #45 OR #46 OR #47 OR #48 OR #49 OR #50 OR #51 OR #52

#54 #23 AND #53

**EMBASE**

#1 'dual antiplatelet therapy'/exp

#2 'dual antiplatelet therapy':ti,ab,kw OR 'antiplatelet combination therapy':ti,ab,kw OR 'combination antiplatelet therapy':ti,ab,kw OR 'dual anti-platelet therapy':ti,ab,kw

#3 'clopidogrel'/exp

#4 clopidogrel:ti,ab,kw OR 'clopidogrel besilate':ti,ab,kw OR 'clopidogrel besylate':ti,ab,kw OR 'clopidogrel bisulfate':ti,ab,kw OR 'clopidogrel bisulphate':ti,ab,kw OR 'clopidogrel bms':ti,ab,kw OR 'clopidogrel hcs':ti,ab,kw OR 'clopidogrel hydrobromide':ti,ab,kw OR 'clopidogrel hydrochloride':ti,ab,kw OR 'clopidogrel hydrogen sulfate':ti,ab,kw OR 'clopidogrel hydrogen sulphate':ti,ab,kw OR 'clopidogrel winthrop':ti,ab,kw OR 'clopilet':ti,ab,kw OR 'grepid':ti,ab,kw OR iscover:ti,ab,kw OR 'pcr 4099':ti,ab,kw OR pcr4099:ti,ab,kw OR plavix:ti,ab,kw OR 'sr 25989':ti,ab,kw OR 'sr 25990c':ti,ab,kw OR sr25989:ti,ab,kw OR sr25990c:ti,ab,kw OR zopya:ti,ab,kw OR zylagren:ti,ab,kw OR zyllt:ti,ab,kw

#5 'ticagrelor'/exp

#6 ticagrelor:ti,ab,kw OR 'azd 6140':ti,ab,kw OR azd6140:ti,ab,kw OR brilinta:ti,ab,kw OR brilique:ti,ab,kw OR possia:ti,ab,kw

#7 'prasugrel'/exp

#8 prasugrel:ti,ab,kw OR 'cs 747':ti,ab,kw OR cs747:ti,ab,kw OR effient:ti,ab,kw OR efient:ti,ab,kw OR 'ly 640315':ti,ab,kw OR 'ly640315':ti,ab,kw OR 'prasugrel benzenesulfonate':ti,ab,kw OR 'prasugrel besilate':ti,ab,kw OR 'prasugrel besylate':ti,ab,kw OR 'prasugrel hcl':ti,ab,kw OR 'prasugrel hydrochloride':ti,ab,kw

#9 'p2y12 antagonist':ti,ab,kw OR 'p2y12 antagonists':ti,ab,kw OR 'p2y12 inhibitor':ti,ab,kw OR 'p2y12 inhibitors':ti,ab,kw OR 'p2y12 receptor antagonist':ti,ab,kw OR 'p2y12 receptor antagonists':ti,ab,kw OR 'p2y12 receptor inhibitor':ti,ab,kw OR 'p2y12 receptor inhibitors':ti,ab,kw OR 'p2y12 inhibition':ti,ab,kw

#10 'percutaneous coronary intervention'/exp

#11 'percutaneous coronary intervention':ti,ab,kw OR 'revascularizations, percutaneous coronary':ti,ab,kw OR 'percutaneous coronary revascularization':ti,ab,kw OR 'coronary interventions, percutaneous':ti,ab,kw OR 'coronary revascularization, percutaneous':ti,ab,kw OR 'coronary intervention, percutaneous':ti,ab,kw OR 'intervention, percutaneous coronary':ti,ab,kw OR 'percutaneous coronary revascularizations':ti,ab,kw OR 'coronary revascularizations, percutaneous':ti,ab,kw OR 'percutaneous coronary interventions':ti,ab,kw OR 'revascularization, percutaneous coronary':ti,ab,kw OR 'interventions, percutaneous coronary':ti,ab,kw

#12 'acute coronary syndrome'/exp

#13 'acute coronary syndrome':ti,ab,kw OR 'acute coronary syndromes':ti,ab,kw OR 'syndrome, acute coronary':ti,ab,kw OR 'coronary syndromes, acute':ti,ab,kw OR 'syndromes, acute coronary':ti,ab,kw OR 'coronary syndrome, acute':ti,ab,kw

#14 dapt:ti,ab,kw

#15 acs:ti,ab,kw

#16 pci:ti,ab,kw

#17 #1 OR #2 OR #3 OR #4 OR #5 OR #6 OR #7 OR #8 OR #9 OR #10 OR #11 OR #12 OR #13 OR #14 OR #15 OR #16

#18 ppi:ti,ab,kw

#19 'proton pump inhibitor'/exp

#20 'proton pump inhibitor':ti,ab,kw OR 'gastric proton pump inhibitor':ti,ab,kw OR 'hydrogen potassium adenosine triphosphatase inhibitor':ti,ab,kw OR 'hydrogen potassium atpase inhibitor':ti,ab,kw OR 'proton pump inhibitors':ti,ab,kw

#21 'omeprazole'/exp

#22 omeprazole:ti,ab,kw OR aleprozil:ti,ab,kw OR antra:ti,ab,kw OR 'antra mups':ti,ab,kw OR arapride:ti,ab,kw OR audazol:ti,ab,kw OR baromezole:ti,ab,kw OR desec:ti,ab,kw OR dolintol:ti,ab,kw OR domer:ti,ab,kw OR dudencer:ti,ab,kw OR duogas:ti,ab,kw OR emeproton:ti,ab,kw OR epirazole:ti,ab,kw OR ezipol:ti,ab,kw OR gasec:ti,ab,kw OR 'gasec gastrocaps':ti,ab,kw OR gastec:ti,ab,kw OR gastop:ti,ab,kw OR gastrimut:ti,ab,kw OR gastrolac:ti,ab,kw OR gastroloc:ti,ab,kw OR glaveral:ti,ab,kw OR 'h 168 68':ti,ab,kw OR 'h 168-68':ti,ab,kw OR 'h etom':ti,ab,kw OR 'h168 68':ti,ab,kw OR hovizol:ti,ab,kw OR hyposec:ti,ab,kw OR inhibitron:ti,ab,kw OR inhipump:ti,ab,kw OR logastric:ti,ab,kw OR lomac:ti,ab,kw OR lopraz:ti,ab,kw OR losamel:ti,ab,kw OR losec:ti,ab,kw OR 'losec mups':ti,ab,kw OR losecosan:ti,ab,kw OR ludea:ti,ab,kw OR madiprazole:ti,ab,kw OR maxor:ti,ab,kw OR medoprazole:ti,ab,kw OR medral:ti,ab,kw OR meiceral:ti,ab,kw OR mepral:ti,ab,kw OR mepzol:ti,ab,kw OR mezzopram:ti,ab,kw OR miol:ti,ab,kw OR miracid:ti,ab,kw OR mopral:ti,ab,kw OR mopralpro:ti,ab,kw OR nocid:ti,ab,kw OR ocid:ti,ab,kw OR ogal:ti,ab,kw OR olexin:ti,ab,kw OR omedar:ti,ab,kw OR omelon:ti,ab,kw OR 'omep uno':ti,ab,kw OR omepral:ti,ab,kw OR omeprazen:ti,ab,kw OR omeprazol:ti,ab,kw OR 'omeprazole magnesium':ti,ab,kw OR 'omeprazole sodium':ti,ab,kw OR omeprazon:ti,ab,kw OR omepril:ti,ab,kw OR omeraz:ti,ab,kw OR omesec:ti,ab,kw OR omestad:ti,ab,kw OR omezin:ti,ab,kw OR omezol:ti,ab,kw OR omezolan:ti,ab,kw OR omezole:ti,ab,kw OR omezzol:ti,ab,kw OR omisec:ti,ab,kw OR omizac:ti,ab,kw OR omolin:ti,ab,kw OR ompranyt:ti,ab,kw OR omprazole:ti,ab,kw OR onexal:ti,ab,kw OR oprax:ti,ab,kw OR ozoken:ti,ab,kw OR parizac:ti,ab,kw OR penrazole:ti,ab,kw OR pepticum:ti,ab,kw OR peptidin:ti,ab,kw OR peptilcer:ti,ab,kw OR peptizole:ti,ab,kw OR 'pra sec':ti,ab,kw OR prazidec:ti,ab,kw OR prazole:ti,ab,kw OR prilosec:ti,ab,kw OR 'prilosec otc':ti,ab,kw OR prisolec:ti,ab,kw OR probitor:ti,ab,kw OR proceptin:ti,ab,kw OR protoloc:ti,ab,kw OR ramezol:ti,ab,kw OR rapinex:ti,ab,kw OR reglacid:ti,ab,kw OR risek:ti,ab,kw OR romep:ti,ab,kw OR roweprazol:ti,ab,kw OR secrepina:ti,ab,kw OR severon:ti,ab,kw OR stomacer:ti,ab,kw OR stomec:ti,ab,kw OR stozole:ti,ab,kw OR suifac:ti,ab,kw OR ulceral:ti,ab,kw OR ulcozol:ti,ab,kw OR ulnor:ti,ab,kw OR ulsek:ti,ab,kw OR ulsen:ti,ab,kw OR ulzol:ti,ab,kw OR vulcasid:ti,ab,kw OR wonmp:ti,ab,kw OR xoprin:ti,ab,kw OR zatrol:ti,ab,kw OR zefxon:ti,ab,kw OR zenpro:ti,ab,kw OR zimor:ti,ab,kw OR zoltum:ti,ab,kw

#23 'esomeprazole'/exp

#24 esomeprazole:ti,ab,kw OR esomeprazol:ti,ab,kw OR 'esomeprazole magnesium':ti,ab,kw OR 'esomeprazole potassium':ti,ab,kw OR 'esomeprazole sodium':ti,ab,kw OR esoprax:ti,ab,kw OR 'h 199 18':ti,ab,kw OR 'h 199-18':ti,ab,kw OR 'h 19918':ti,ab,kw OR 'h199 18':ti,ab,kw OR 'h19918':ti,ab,kw OR inexium:ti,ab,kw OR nexium:ti,ab,kw OR 'nexium 24hr':ti,ab,kw OR 'nexium control':ti,ab,kw OR 'nexium iv':ti,ab,kw OR 'nexium mups':ti,ab,kw OR perprazole:ti,ab,kw OR sompraz:ti,ab,kw

#25 'pantoprazole'/exp

#26 pantoprazole:ti,ab,kw OR anagastra:ti,ab,kw OR branzol:ti,ab,kw OR 'by 1023':ti,ab,kw OR by1023:ti,ab,kw OR controloc:ti,ab,kw OR 'controloc control':ti,ab,kw OR eupantol:ti,ab,kw OR inipom:ti,ab,kw OR inipomp:ti,ab,kw OR pantecta:ti,ab,kw OR 'pantecta control':ti,ab,kw OR pantodac:ti,ab,kw OR pantodar:ti,ab,kw OR pantoloc:ti,ab,kw OR 'pantoloc control':ti,ab,kw OR pantop:ti,ab,kw OR 'pantoprazole sodium':ti,ab,kw OR 'pantoprazole sodium sesquihydrate':ti,ab,kw OR pantozol:ti,ab,kw OR 'pantozol control':ti,ab,kw OR pepticus:ti,ab,kw OR protium:ti,ab,kw OR protonix:ti,ab,kw OR 'protonix iv':ti,ab,kw OR rifun:ti,ab,kw OR 'rifun 40':ti,ab,kw OR 'skf 96022':ti,ab,kw OR skf96022:ti,ab,kw OR somac:ti,ab,kw OR 'somac control':ti,ab,kw OR ulcepraz:ti,ab,kw OR ulcotenal:ti,ab,kw OR ziprol:ti,ab,kw OR zurcal:ti,ab,kw OR zurcale:ti,ab,kw OR zurcazol:ti,ab,kw

#27 'rabeprazole'/exp

#28 rabeprazole:ti,ab,kw OR aciphex:ti,ab,kw OR 'aciphex sprinkle':ti,ab,kw OR 'dexrabeprazole':ti,ab,kw OR 'ly 307640':ti,ab,kw OR ly307640:ti,ab,kw OR pariet:ti,ab,kw OR pariprazole:ti,ab,kw OR 'pariprazole sodium':ti,ab,kw OR rabec:ti,ab,kw OR rabeloc:ti,ab,kw OR 'rabeprazole sodium':ti,ab,kw

#29 'lansoprazole'/exp

#30 lansoprazole:ti,ab,kw OR 'a 65006':ti,ab,kw OR a65006:ti,ab,kw OR 'abt 006':ti,ab,kw OR abt006:ti,ab,kw OR 'ag 1749':ti,ab,kw OR ag1749:ti,ab,kw OR agopton:ti,ab,kw OR bamalite:ti,ab,kw OR banilux:ti,ab,kw OR betalans:ti,ab,kw OR compraz:ti,ab,kw OR daxar:ti,ab,kw OR dostab:ti,ab,kw OR duomate:ti,ab,kw OR ilsatec:ti,ab,kw OR inhipraz:ti,ab,kw OR keval:ti,ab,kw OR lancid:ti,ab,kw OR lancopen:ti,ab,kw OR langaton:ti,ab,kw OR lanpra:ti,ab,kw OR lanpraz:ti,ab,kw OR lanprol:ti,ab,kw OR lanproton:ti,ab,kw OR lansazol:ti,ab,kw OR lansobene:ti,ab,kw OR lansol:ti,ab,kw OR lansone:ti,ab,kw OR lansop:ti,ab,kw OR lansopep:ti,ab,kw OR lansoprazol:ti,ab,kw OR lansox:ti,ab,kw OR lansozole:ti,ab,kw OR lanster:ti,ab,kw OR lanston:ti,ab,kw OR lanvell:ti,ab,kw OR lanximed:ti,ab,kw OR lanzo:ti,ab,kw OR 'lanzol 30':ti,ab,kw OR lanzopral:ti,ab,kw OR lanzoprazole:ti,ab,kw OR lanzor:ti,ab,kw OR lanzul:ti,ab,kw OR lapraz:ti,ab,kw OR laprazol:ti,ab,kw OR laproton:ti,ab,kw OR lasgan:ti,ab,kw OR limpidex:ti,ab,kw OR lopral:ti,ab,kw OR monolitum:ti,ab,kw OR ogast:ti,ab,kw OR ogasto:ti,ab,kw OR ogastoro:ti,ab,kw OR ogastro:ti,ab,kw OR opiren:ti,ab,kw OR pampe:ti,ab,kw OR praton:ti,ab,kw OR prevacid:ti,ab,kw OR 'prevacid 24 hr':ti,ab,kw OR 'prevacid fastab':ti,ab,kw OR 'prevacid iv':ti,ab,kw OR 'prevacid solutab':ti,ab,kw OR prezal:ti,ab,kw OR prolanz:ti,ab,kw OR prosogan:ti,ab,kw OR pysolan:ti,ab,kw OR 'sopralan 30':ti,ab,kw OR suprecid:ti,ab,kw OR takepron:ti,ab,kw OR 'takepron od':ti,ab,kw OR tanzolan:ti,ab,kw OR ulpax:ti,ab,kw OR zoton:ti,ab,kw OR 'zoton fastab':ti,ab,kw

#31 'dexlansoprazole'/exp

#32 dexlansoprazole:ti,ab,kw OR dexilant:ti,ab,kw OR 'dexilant solutab':ti,ab,kw OR kapidex:ti,ab,kw OR 't 168390':ti,ab,kw OR t168390:ti,ab,kw OR 'tak 390':ti,ab,kw OR 'tak 390mr':ti,ab,kw OR tak390:ti,ab,kw OR tak390mr:ti,ab,kw

#33 'ilaprazole'/exp

#34 ilaprazole:ti,ab,kw OR 'iy 81149':ti,ab,kw OR iy81149:ti,ab,kw

#35 'leminoprazole'/exp

#36 leminoprazole:ti,ab,kw OR leminon:ti,ab,kw OR 'nc 1300 03':ti,ab,kw OR 'nc 1300 o 3':ti,ab,kw

#37 'picoprazole'/exp

#38 picoprazole:ti,ab,kw OR 'h 149-94':ti,ab,kw

#39 'pumaprazole'/exp

#40 pumaprazole:ti,ab,kw OR 'by 841':ti,ab,kw OR by841:ti,ab,kw

#41 'timoprazole'/exp

#42 'saviprazole'/exp

#43 saviprazole:ti,ab,kw OR hoe731:ti,ab,kw OR 'hoe 731':ti,ab,kw

#44 'benatoprazole'/exp

#45 benatoprazole:ti,ab,kw OR protop:ti,ab,kw OR tenatoprazole:ti,ab,kw OR 'tu 199':ti,ab,kw OR tu199:ti,ab,kw OR ulsacare:ti,ab,kw

#46 #18 OR #19 OR #20 OR #21 OR #22 OR #23 OR #24 OR #25 OR #26 OR #27 OR #28 OR #29 OR #30 OR #31 OR #32 OR #33 OR #34 OR #35 OR #36 OR #37 OR #38 OR #39 OR #40 OR #41 OR #42 OR #43 OR #44 OR #45

#47 #17 AND #46

**WEB OF SCIENCE**

#1 TOPIC: (Dual Anti-Platelet Therapy) OR TOPIC: (Dual AntiPlatelet Therapy) OR TOPIC: (Anti-Platelet Therapies, Dual) OR TOPIC: (Anti-Platelet Therapy, Dual) OR TOPIC: (Dual Anti-Platelet Therapies) OR TOPIC: (Dual Anti Platelet Therapy)

#2 TOPIC: (Clopidogrel) OR TOPIC: (SC 25989C) OR TOPIC: (SC 25990C) OR TOPIC: (SR 25989) OR TOPIC: (Clopidogrel-Mepha) OR TOPIC: (Clopidogrel Mepha) OR TOPIC: (Clopidogrel Sandoz) OR TOPIC: (Iscover) OR TOPIC: (Clopidogrel Napadisilate) OR TOPIC: (Clopidogrel Hydrochloride) OR TOPIC: (PCR 4099) OR TOPIC: (PCR-4099) OR TOPIC: (Clopidogrel Besylate) OR TOPIC: (Clopidogrel Besilate) OR TOPIC: (Clopidogrel, (+)(S)-isomer) OR TOPIC: (Plavix) OR TOPIC: (Clopidogrel Bisulfate)

#3 TOPIC: (Ticagrelor) OR TOPIC: (Brilique) OR TOPIC: (AZD 6140) OR TOPIC: (AZD6140) OR TOPIC: (AZD-6140) OR TOPIC: (Brilinta) OR TOPIC (3-(7-((2-(3,4-Difluorophenyl)cyclopropyl)amino)-5-(propylthio)-3H-(1-3)-triazolo(4,5-d)pyrimidin-3-yl)-5-(2-hydroxyethoxy)cyclopentane-1,2-dio)

#4 TOPIC: (Prasugrel Hydrochloride) OR TOPIC: (Hydrochloride, Prasugrel) OR TOPIC: (Prasugrel HCl) OR TOPIC: (HCl, Prasugrel) OR TOPIC: (CS 747) OR TOPIC: (747, CS) OR TOPIC: (CS-747) OR TOPIC: (CS747) OR TOPIC: (Prasugrel) OR TOPIC: (Efient) OR TOPIC: (Effient) OR TOPIC: (LY 640315) OR TOPIC: (640315, LY) OR TOPIC: (LY640315) OR TOPIC: (LY-640315)

#5 TOPIC: (P2Y12 antagonist) OR TOPIC: (P2Y12 antagonists) OR TOPIC: (P2Y12 inhibitor) OR TOPIC: (P2Y12 inhibitors) OR TOPIC: (P2Y12 receptor antagonist) OR TOPIC题: (P2Y12 receptor antagonists) OR TOPIC: (P2Y12 receptor inhibitor) OR TOPIC: (P2Y12 receptor inhibitors) OR TOPIC: (P2Y12 Inhibition)

#6 TOPIC: (Percutaneous Coronary Intervention) OR TOPIC: (Coronary Intervention, Percutaneous) OR TOPIC: (Coronary Interventions, Percutaneous) OR TOPIC: (Intervention, Percutaneous Coronary) OR TOPIC: (nterventions, Percutaneous Coronary) OR TOPIC: (Percutaneous Coronary Interventions) OR TOPIC: (Percutaneous Coronary Revascularization) OR TOPIC: (Coronary Revascularization, Percutaneous) OR TOPIC: (Coronary Revascularizations, Percutaneous) OR TOPIC: (Percutaneous Coronary Revascularizations) OR TOPIC: (Revascularization, Percutaneous Coronary) OR TOPIC: (Revascularizations, Percutaneous Coronary)

#7 TOPIC: (Acute Coronary Syndrome) OR TOPIC: (Acute Coronary Syndromes) OR TOPIC: (Coronary Syndrome, Acute) ORTOPIC: (Coronary Syndromes, Acute) OR TOPIC: (Syndrome, Acute Coronary) OR TOPIC: (Syndromes, Acute Coronary)

#8 TOPIC: (DAPT)

#9 TOPIC: (ACS)

#10 TOPIC: (PCI)

#11 #10 OR #9 OR #8 OR #7 OR #6 OR #5 OR #4 OR #3 OR #2 OR #1

#12 TOPIC: (PPI)

#13 TOPIC: (Proton Pump Inhibitors) OR TOPIC: (Inhibitors, Proton Pump) OR TOPIC: (Proton Pump Inhibitor)

#14 TOPIC: (Omeprazole) OR TOPIC: (Prilosec) OR TOPIC: (Omeprazole Sodium) OR TOPIC: (Sodium, Omeprazole) OR TOPIC: (H 168-68) OR TOPIC: (H 168 68) OR TOPIC: (H 16868) OR TOPIC: (Omeprazole Magnesium) OR TOPIC: (Magnesium, Omeprazole)

#15 TOPIC: (Esomeprazole) OR TOPIC: (Esomeprazole Strontium) OR TOPIC: (Strontium, Esomeprazole) OR TOPIC: (Esomeprazole Magnesium) OR TOPIC: (Nexium) OR TOPIC: (Esomeprazole Potassium) OR TOPIC: (Esomeprazole Strontium Anhydrous) OR TOPIC: (Esomeprazole Sodium)

#16 TOPIC: (Pantoprazole) OR TOPIC: (Pantoprazole Sodium) OR TOPIC: (SK AND F 96022) OR TOPIC: (SKF-96022) OR TOPIC: (SKF 96022) OR TOPIC: (SKF96022) OR TOPIC: (SK AND F-96022) OR TOPIC: (SK AND F96022) OR TOPIC: (BY 1023) OR TOPIC: (BY-1023) OR TOPIC: (BY1023) OR TOPIC: (Protonix)

#17 TOPIC: (Rabeprazole) OR TOPIC: (2-((4-(3-methoxypropoxy)-3-methylpyridin-2-yl)methylsulfinyl)-1H-benzimidazole) OR TOPIC: (Dexrabeprazole) OR TOPIC: (E 3810) OR TOPIC: (E3810) OR TOPIC: (Pariet) OR TOPIC: (Rabeprazole Sodium) OR TOPIC: (Sodium, Rabeprazole) OR TOPIC: (1H-Benzimidazole, 2-(((4-(3-methoxypropoxy)-3-methyl-2-pyridinyl)methyl)sulfinyl)-, Sodium Salt) OR TOPIC: (Aciphex) OR TOPIC: (LY-307640) OR TOPIC: (LY 307640) OR TOPIC: (LY307640)

#18 TOPIC: (Lansoprazole) OR TOPIC: (Lansoprazol) OR TOPIC: (2-(((3-Methyl-4-(2,2,2-trifluoroethoxy)-2-pyridyl)methyl)sulfinyl)benzimidazole) OR TOPIC: (Lansoprazoles) OR TOPIC: (Ogastro) OR TOPIC: (AG 1749) OR TOPIC: (AG-1749) OR TOPIC: (AG1749) OR TOPIC: (Agopton) OR TOPIC: (Bamalite) OR TOPIC: (Lansol) OR TOPIC: (LansoprazoleSodium) OR TOPIC: (Sodium,Lansoprazole) OR TOPIC: (Lanzor) OR TOPIC: (Monolitum) OR TOPIC: (Opiren) OR TOPIC: (Prevacid) OR TOPIC: (Pro Ulco) OR TOPIC: (Promeco) OR TOPIC: (Takepron) OR TOPIC: (Ulpax) OR TOPIC: (Zoton) OR TOPIC: (Ogast) OR TOPIC: (Prezal)

#19 TOPIC: (Dexlansoprazole) OR TOPIC: (Lansoprazole, R-Isomer) OR TOPIC: (Lansoprazole, R Isomer) OR TOPIC: (R-Isomer Lansoprazole) OR TOPIC: (2-((R)-((3-Methyl-4-(2,2,2-trifluoroethoxy)-2-pyridinyl)methyl)sulfinyl)-1H-benzimidazole) OR TOPIC: (R-Lansoprazole) OR TOPIC: (R Lansoprazole) OR TOPIC: (Dexlansoprazole Sesquihydrate) OR TOPIC: (AK 390MR) OR TOPIC: (TAK390MR) OR TOPIC: (TAK-390MR) OR TOPIC: (TAK-390) OR TOPIC: (TAK 390) OR TOPIC: (TAK390) OR TOPIC: (Dexilant) OR TOPIC: (T-168390) OR TOPIC: (T 168390) OR TOPIC: (T168390)

#20 TOPIC: (ilaprazole) OR TOPIC: (IY 81149) OR TOPIC: (IY81149) OR TOPIC: (IY-81149) OR TOPIC: (leminoprazole) OR TOPIC: (NC 1300-O-3) OR TOPIC: (NC-1300-O-3) OR TOPIC: (picoprazole) OR TOPIC: (2-((2-(3-methyl)pyridyl-methyl)sulfinyl)-5-methoxycarbonyl-6-methylbenzimidazole) OR TOPIC: (H 149-94) OR TOPIC: (pumaprazole) OR TOPIC: (timoprazole) OR TOPIC: (2-((2-pyridylmethyl)sulfinyl)benzimidazole) OR TOPIC: (saviprazole) OR TOPIC: (tenatoprazole) OR TOPIC: (5-methyl-2((4-methoxy-3,5-dimethyl-2-pyridyl)methyl)sulfinyl-imidazo(4,5-b)pyridine) OR TOPIC: (5-methoxy-2-(((4-methoxy-3,5-dimethylpyrid-2-yl)methyl)sulfinyl)-1H-imidazo(4,5-b)pyridine) OR TOPIC: (TU 199) OR TOPIC: (TU199) OR TOPIC: (TU-199)

#21 #20 OR #19 OR #18 OR #17 OR #16 OR #15 OR #14 OR #13 OR #12

#22 #21 AND #11
