# Supplementary material for: Clinical Outcomes of Concomitant Use of Proton Pump Inhibitors and Dual Antiplatelet Therapy: A Systematic Review and Meta-Analysis
Source: Front Pharmacol. 2021 Aug 2;12:694698. doi: 10.3389/fphar.2021.694698 (PMC8366318; doi:10.3389/fphar.2021.694698)
Supplement: Supplementary file 3 [file Table5.DOCX]

**Supplement 5. Sensitivity analysis**

| Drug | Subgorup | Studies (year) | Pooled Effect Size (95% CI) | I^2^ statistic |
| --- | --- | --- | --- | --- |
| Omeprazole | Hazard ratio only | Kreutz (2010)  O'Donoghue(2009)  Ray (2010)  Schmidt (2012)  Simon (2011) | 1.00 (0.76–1.32) | 0% |
|  | One-year follow up | Kreutz (2010)  Ray (2010)  Schmidt (2012)  Simon (2011) | 1.03 (0.74–1.43) |  |
| Esomeprazole | Hazard ratio only | Kreutz (2010)  O'Donoghue(2009)  Ray (2010)  Schmidt (2012)  Simon (2011) | 1.16 (0.87–1.54) | 0% |
|  | One-year follow up | Kreutz (2010)  Ray (2010)  Schmidt (2012)  Simon (2011) | 1.17 (0.84-1.64) |  |
| Lansoprazole | Hazard ratio only | Kreutz (2010)  O'Donoghue(2009)  Ray (2010)  Schmidt (2012)  Simon (2011) | 1.24 (1.07–1.45) | 0% |
|  | One-year follow up | Kreutz (2010)  Ray (2010)  Schmidt (2012)  Simon (2011) | 1.28 (1.08–1.50) |  |
| Pantoprazole | Hazard ratio only | Kreutz (2010)  O'Donoghue(2009)  Ray (2010)  Schmidt (2012)  Simon (2011) | 1.31 (1.01–1.70) | 0% |
|  | One-year follow up | Kreutz (2010)  Ray (2010)  Schmidt (2012)  Simon (2011) | 1.43 (1.11–1.84) |  |
| Rabeprazole | Hazard ratio only | Hokimoto (2014)  Ray (2010) | 0.61 (0.36-1.03) | 0% |

CI, confidence interval.
