# Supplementary material for: Clinical Outcomes of Concomitant Use of Proton Pump Inhibitors and Dual Antiplatelet Therapy: A Systematic Review and Meta-Analysis
Source: Front Pharmacol. 2021 Aug 2;12:694698. doi: 10.3389/fphar.2021.694698 (PMC8366318; doi:10.3389/fphar.2021.694698)
Supplement: Supplementary file 4 [file Table3.DOCX]

**Supplement 3. Definition of outcomes**

| Study | Definition of MACEs | Definition of gastrointestinal bleeding or upper gastrointestinal bleeding |
| --- | --- | --- |
| Bhatt (2010) | CV death, nonfatal MI, coronary revascularization, ischemic stroke | Overt bleeding of gastroduodenal origin (confirmed by means of upper endoscopy or radiography), overt UGI bleeding of unknown origin, bleeding of presumed occult GI origin with a documented decrease in hemoglobin of 2g per deciliter or more or in the hematocrit by 10% or more from the baseline value |
| Gao (2009) | NA | NR |
| Goodman (2012) | CV death, MI, stroke | NA |
| Jensen (2017) | Unstable angina pectoris, MI and all-cause mortality | Hematemesis or melena, history of hematemesis, melena and low hemoglobin levels or need for blood transfusion |
| Ren (2011) | NA | NR |
| Wu (2011) | NA | Hematemesis, nasogastric aspirate containing blood or coffee-grounds material, melena, hematochezia |
| Zhang (2019) | Recurrent thrombosis in stents, recurrent MI, revascularization, malignant arrhythmia, cerebral infarction, cardiac death | NR |
| Charlot (2010) | NA | NR |
| Ho (2009) | All-cause mortality, MI, unstable angina | NA |
| Hoedemaker (2018) | NA | NR |
| Hokimoto (2014) | CV death, nonfatal MI, unstable angina, ischemic stroke, or coronary revascularization to new lesions | NR |
| Jiang (2013) | NA | Significant occult gastrointestinal bleeding was defined as a blood hemoglobin fall ≥ 2 g/dL with no identifiable extraintestinal source |
| Juurlink (2009) | NA | NA |
| Kreutz (2010) | Hospitalization for a cerebrovascular  event (stroke or transient ischemic attack), ACS, coronary, CV death | NA |
| Ng (2008) | NA | Significant occult gastrointestinal bleeding was defined as a hemoglobin drop of more than or equal to 2 g/dL without an identifiable extraintestinal  source |
| O'Donoghue (2009) | CV death, MI, stroke | NA |
| Rassen (2009) | MI hospitalization, death | NA |
| Ray (2010) | MI, sudden cardiac death, stroke, CV death | Bleed at a gastroduodenal site (exclude angiodysplasia of the stomach or duodenum) |
| Schmidt (2012) | MI, stroke, stent thrombosis, target lesion revascularization, cardiac death | NA |
| Sehested (2019) | NA | Hospital admission or death from primary diagnoses denoting bleeding (bleeding ulcer, haematemesis, haemorrhagic gastritis and duodenitis, oesophageal  varices with bleeding, and Mallory Weiss bleeding). |
| Simon (2011) | Death, MI, stroke | NA |
| Yan (2016) | All-cause death, MI | NA |

ACS, acute coronary syndrome; CV, cardiovascular; GI, gastrointestinal; MACE, major adverse cardiac events; MI, myocardial infarction; NA, not applied; NR, not reported; UGI, upper gastrointestinal;
