# Supplementary material for: Clinical Outcomes of Concomitant Use of Proton Pump Inhibitors and Dual Antiplatelet Therapy: A Systematic Review and Meta-Analysis
Source: Front Pharmacol. 2021 Aug 2;12:694698. doi: 10.3389/fphar.2021.694698 (PMC8366318; doi:10.3389/fphar.2021.694698)
Supplement: Supplementary file 5 [file Table4.DOCX]

**Supplement 4. Risk of bias of included studies**

**Randomised controlled trials: Cochrane risk assessment tool**

| **Study(year)** | **Random sequence generation** | **Allocation concealment** | **Blinding of participants and personnel** | **Blinding of outcome assessment** | **Incomplete outcome data** | **Selective reporting** | **Other sources of bias** |
| --- | --- | --- | --- | --- | --- | --- | --- |
| Bhatt(2010) | Low risk | Low risk | Low risk | Low risk | Low risk | Low risk | Low risk |
| Gao(2009) | Unclear Risk | Unclear Risk | Low risk | Unclear Risk | Low risk | Low risk | Low risk |
| Jensen(2017) | Low risk | Low risk | High risk | Low risk | Low risk | Low risk | Low risk |
| Ren(2011) | Unclear Risk | Unclear Risk | Unclear Risk | Unclear Risk | Low risk | High risk | Low risk |
| Wu(2011) | Low risk | Unclear Risk | Low risk | Low risk | Low risk | Low risk | Low risk |
| Zhang(2019) | Unclear Risk | Unclear Risk | Low risk | Unclear Risk | Low risk | Low risk | Low risk |

**Cohort studies: Newcastle-Ottawa quality assessment scale**

| **Reference (year)** | **Selection** | | | | **Comparability** | **Intervention/Exposure** | | | **Total** |
| --- | --- | --- | --- | --- | --- | --- | --- | --- | --- |
|  | **Representative of exposed cohort** | **Selection of non- exposed cohort** | **Ascertainmet of exposure** | **Outcome not at start** | **Comparability** | **Assessment of outcome** | **Length of follow-up** | **Adequacy of follow-up** |  |
| Charlot (2010) | 1 | 1 | 1 | 1 | 2 | 1 | 1 | 1 | 9 |
| Goodman (2012) | 1 | 1 | 1 | 1 | 2 | 1 | 1 | 1 | 9 |
| Ho (2009) | 1 | 1 | 1 | 1 | 2 | 1 | 1 | 1 | 9 |
| Hoedemaker (2018) | 1 | 1 | 1 | 1 | 2 | 1 | 1 | 1 | 9 |
| Hokimoto (2014) | 1 | 1 | 1 | 1 | 2 | 1 | 1 | 1 | 9 |
| Kreutz (2010) | 1 | 1 | 1 | 1 | 2 | 1 | 1 | 1 | 9 |
| Ng (2008) | 1 | 1 | 1 | 1 | 2 | 1 | 1 | 1 | 9 |
| O'Donoghue (2009) | 1 | 1 | 1 | 1 | 2 | 0 | 1 | 1 | 8 |
| Rassen (2009) | 1 | 1 | 1 | 1 | 2 | 1 | 1 | 1 | 9 |
| Ray (2010) | 1 | 1 | 1 | 1 | 2 | 1 | 1 | 1 | 9 |
| Schmidt (2012) | 1 | 1 | 1 | 1 | 2 | 1 | 1 | 1 | 9 |
| Sehested (2019) | 1 | 1 | 1 | 1 | 2 | 1 | 1 | 1 | 9 |
| Simon (2011) | 1 | 1 | 1 | 1 | 2 | 0 | 1 | 1 | 8 |
| Yan (2016) | 1 | 1 | 1 | 1 | 2 | 0 | 1 | 1 | 8 |

**Case-control studies: Newcastle-Ottawa quality assessment scale**

| **Study (year)** | **Selection** | | | | **Comparability** | **Intervention/Exposure** | | | **Total** |
| --- | --- | --- | --- | --- | --- | --- | --- | --- | --- |
|  | **Case definition adequate** | **Representative of cases** | **Selection of controls** | **Definition of controls** | **Comparability** | **Ascertainment of exposure** | **Same method of ascertainment for cases and controls** | **Non-response rate** |  |
| Jiang (2013) | 1 | 1 | 0 | 1 | 2 | 1 | 1 | 1 | 8 |
| Juurlink (2009) | 1 | 1 | 0 | 1 | 2 | 1 | 1 | 1 | 8 |
